# Transplanting Human Neural Stem Cells with ≈50% Reduction of *SOX9* Gene Dosage Promotes Tissue Repair and Functional Recovery from Severe Spinal Cord Injury

**DOI:** 10.1002/advs.202205804

**Published:** 2023-06-09

**Authors:** Jessica Aijia Liu, Kin Wai Tam, Yong Long Chen, Xianglan Feng, Christy Wing Lam Chan, Amos Lok Hang Lo, Kenneth Lap‐Kei Wu, Man‐Ning Hui, Ming‐Hoi Wu, Ken Kwok‐Keung Chan, May Pui Lai Cheung, Chi Wai Cheung, Daisy Kwok‐Yan Shum, Ying‐Shing Chan, Martin Cheung

**Affiliations:** ^1^ Department of Anaesthesiology School of Clinical Medicine Li Ka Shing Faculty of Medicine The University of Hong Kong Hong Kong China; ^2^ Department of Neuroscience Tat Chee Avenue City University of Hong Kong Hong Kong China; ^3^ School of Biomedical Sciences Li Ka Shing Faculty of Medicine The University of Hong Kong Hong Kong China

**Keywords:** human neural stem cells, motor neurons, pluripotent stem cells, *SOX9*, spinal cord injury

## Abstract

Neural stem cells (NSCs) derived from human pluripotent stem cells (hPSCs) are considered a major cell source for reconstructing damaged neural circuitry and enabling axonal regeneration. However, the microenvironment at the site of spinal cord injury (SCI) and inadequate intrinsic factors limit the therapeutic potential of transplanted NSCs. Here, it is shown that half dose of *SOX9* in hPSCs‐derived NSCs (hNSCs) results in robust neuronal differentiation bias toward motor neuron lineage. The enhanced neurogenic potency is partly attributed to the reduction of glycolysis. These neurogenic and metabolic properties retain after transplantation of hNSCs with reduced *SOX9* expression in a contusive SCI rat model without the need for growth factor‐enriched matrices. Importantly, the grafts exhibit excellent integration properties, predominantly differentiate into motor neurons, reduce glial scar matrix accumulation to facilitate long‐distance axon growth and neuronal connectivity with the host as well as dramatically improve locomotor and somatosensory function in recipient animals. These results demonstrate that hNSCs with half *SOX9* gene dosage can overcome extrinsic and intrinsic barriers, representing a powerful therapeutic potential for transplantation treatments for SCI.

## Graphical abstract

Spinal cord injury (SCI) causes nerve damage and the injury environment favors *SOX9* expression, inhibiting neurogenesis and promoting astrocytic scar formation. Grafting of hNSCs with a half dose of *SOX9* acquires unique intrinsic capacity resulting in enhanced neurogenesis, long axonal growth, and establishing neuronal connectivity with the host that restores locomotor activity and somatosensory function of the SCI animals.

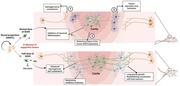

## 1 Introduction

Traumatic spinal cord injury (SCI) causes progressive neuronal loss and axonal damage, leading to defective locomotion and somatosensory function. The ineffectiveness of current clinical management and treatment regimens can leave SCI patients suffering from lifelong disabilities. During the chronic phase of SCI, lesion remodeling leads to the formation of cystic cavitation surrounded by glial scar which contains reactive astrocytes as the major component of a barrier‐like structure to prevent neuronal regeneration. In response to lesion healing, the scar can prevent inflammation from spreading and causing further damage. However, spinal composition and architectures within and around the scar cannot be restored due to low intrinsic regenerative ability of neurons in the adult mammalian central nervous system(CNS) and the post‐injury environment.^[^ ^1^ ^]^ Transplantation of neural stem cells (NSCs) derived from human pluripotent stem cells (hPSCs) at SCI sites has been considered a promising strategy to compensate for the loss of spinal neurons and enable their connectivity with host neurons to restore spinal dysfunctions.^[^ ^2^ ^]^ However, a hostile microenvironment and deficiency of growth factors in the injured spinal cord limited the therapeutic effects of grafted NSCs that are largely determined by their survival, neurogenic potency, integration capacity, and axial identity. In clinically relevant contusive injury models, the injured niche promotes reactive astrogliosis and the differentiation of transplanted NSCs into astrocytes.^[^ ^1^, ^3^ ^]^ Although NSCs embedded with matrices containing growth factor cocktails are used for grafting, protracted time course of human cell differentiation and maturation with less amount of differentiated neuronal subtypes generated results in a limited degree of functional recovery in SCI models.^[^ ^2^, ^3^, ^4^ ^]^ Remodelling hNSC grafts to overcome both extrinsic and intrinsic barriers may provide a more effective repair process for traumatic SCI.

As a member of SOX family of transcription factors, *SOX9* plays a critical role in maintaining NSC multipotentiality. In the conditional knockout of *SOX9* in the ventricular zone where NSCs reside, there was a transient increase in motor neuron formation at the expense of astroglial cells,^[^ ^5^ ^]^ suggesting that *SOX9* is required for the suppression of neurogenesis and glial fate specification. Notably, activated *SOX9* expression is highly associated with the pathogenesis of SCI. Upregulation of *SOX9* was detected in scar‐forming astrocytes around the lesion site following SCI.^[^ ^6^ ^]^ In contrast, global ablation of the *SOX9* gene in adult mice resulted in reduced expression of chondroitin sulfate proteoglycans (CSPGs) that are inhibitors of axonal growth, deposition of extracellular matrix, and expression of GFAP, a marker of astrocyte activation, at the lesion center^[^ ^7^ ^]^ that were accompanied by increased axonal sprouting distal to the lesion, and most importantly, improved recovery of locomotor function following SCI.^[^ ^8^ ^]^ In addition, constitutive activation of *SOX9* in developing neural tubes increased the apoptosis of neural progenitors and neurons, indicating that the prolonged expression of *SOX9* was incompatible with the progression of neurogenesis.^[^ ^9^ ^]^ Indeed, we found that *SOX9* expression gradually diminished during neuronal differentiation of hNSCs in vitro, whereas the injured niche following contusion SCI resulted in upregulated *SOX9* expression in reactive astrocytes and prolonged expression of *SOX9* favors astrocytic differentiation of grafted hNSCs.^[^ ^3c^ ^]^ These findings suggest that persistent expression of *SOX9* prevents the de novo neuronal regeneration process in the injured spinal cord, underlying the possibility of targeting *SOX9* to generate hNSCs with enhanced neurogenic potency and insusceptibility to the hostile milieu for treating SCI.

To recapitulate the gradual diminishment of *SOX9* expression in hNSCs undergoing neuronal differentiation, we carried out shRNA knockdown (KD) of *SOX9* expression in hNSCs via doxycycline‐inducible lentiviral vector in a dose‐dependent manner. We found a reduced level of *SOX9* expression by ≈50% led to precocious neuronal differentiation bias toward spinal motor neuron lineage compared to the scramble control. Further reduction of *SOX9* expression resulted in compromised cell survival and renewal. The enhanced neurogenesis of *SOX9 KD* hNSCs was found to be linked with decreased glycolytic metabolism.^[^ ^10^ ^]^ Recent studies have revealed that neuronal differentiation or/and regeneration requires metabolic remodeling from glycolysis to oxidative phosphorylation. It has been shown that neuronal differentiation from hNSCs requires decreased aerobic glycolysis, whereas constitutive activation of glycolytic genes in hNSCs resulted in robust astrocytes formation and apoptosis of neurons.^[^ ^10^ ^]^ Consistently, enhancing mitochondrial function could promote axonal regeneration and functional restoration after SCI.^[^ ^11^ ^]^ In addition, a few glycolytic enzymes such as TPI‐1 (Triosephosphate isomerase 1), and ENO2 (Enolase) have been identified as SCI biomarkers to correlate with disease progression in moderate/severe SCI models or patients.^[^ ^12^ ^]^ Our data further confirmed that modulating glycolytic activity of NSCs recaptures a dose‐dependent manner of *SOX9* in regulating NSCs renewal, survival, and differentiation. The changes in potency and metabolic state were retained in the *SOX9 KD* hNSCs upon transplantation at the site of contusive SCI in rats that could contribute to their excellent engraftment properties without needing a supportive matrix or growth factor supplementation. The *SOX9 KD* hNSCs were broadly distributed across the lesion site, able to generate substantial amounts of mature neuronal subtypes, and had extended long‐distance axonal outgrowth that formed new relay circuits and synaptic connections with the local sensory‐motor neural networks. In addition, the *SOX9 KD* grafts exerted beneficial effects in ameliorating glial scarring, which further facilitated neuronal outgrowth and innervation of descending host neuronal fibers into the grafts. The combined intrinsic properties and extrinsic effects of *SOX9 KD* grafts enhanced the repair of spinal cord lesions in a post‐injury hostile milieu and effectively improved locomotor and somatosensory deficits. Our findings represent a new paradigm in generating genetically modified hNSCs for the treatment of SCI.

## 2 Results

### 2.1 Injured Microenvironment Induces *SOX9* Expressing Cells and Loss of *SOX9* Expression in Human Neural Stem Cells (hNSCs) is Associated with Neuronal Fate Acquisition

**Figure 1:**
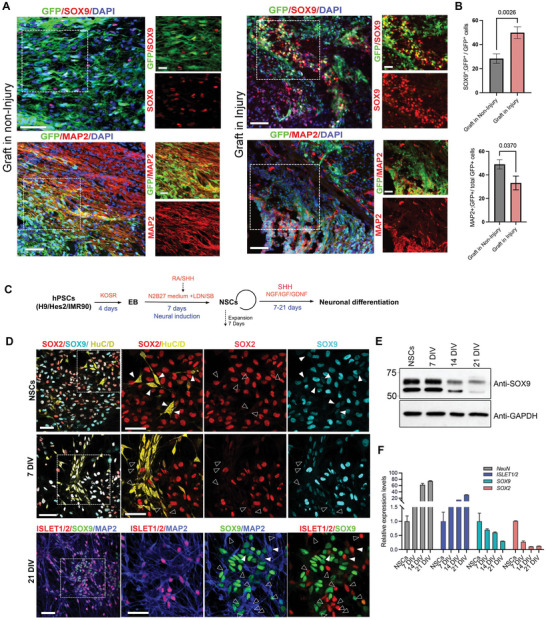
Gradual loss of *SOX9* expression in hNSCs is associated with the acquisition of neuronal fate. A) Representative images showing *SOX9* and MAP2 expressions in GFP^+^ grafts in the non‐injury and injured spinal cords at 2 months after grafting. The white boxes show a magnified view with the indicated markers (scale bar for the magnified view, 200 µm; scale bar for the lower power view, 50 µm). B) Quantification of *SOX9* ^+^ or MAP^+^ cells in GFP^+^ grafts from non‐injury and injured spinal cords. *n* = 5, 3–4 sections/rat, Student's t‐test. C) Overview of the neural induction and neuronal differentiation protocol from human pluripotent stem cells (hPSCs). D) Representative immunofluorescence images for *SOX2*, *SOX9*, and *HuC/D* in hNSCs after 7 days differentiation (DIV) and for *MAP2*, *SOX9*, and ISLET1/2 after 21 days differentiation. White arrowheads indicate a moderate reduction of *SOX9* during neuronal differentiation. The empty arrow indicates differentiating neuronal cells without SOX2 expression but exhibits a moderate reduction of *SOX9* (scale bar, 50 µm). Three independent experiments. E) Representative immunoblot images showing time course analysis of *SOX9* expressions in hNSCs and neuronal differentiation. Three independent experiments. F) Time course analysis of neuronal markers *NeuN* and *ISL1/2*, *SOX2*, and *SOX9* expressions by qPCR. Three independent experiments. All data are expressed as Mean ± SEM.

Previous studies have observed that the expression of *SOX9* derived from ependymal cells contributes to the glial scar in the transected spinal cord.^[^ ^13^ ^]^ To re‐examine the evidence in a more clinically relevant model, we generated a rat spinal contusion model that mimics the most common clinical presentations of a fracture dislocation in humans.^[^ ^14^ ^]^ Indeed, 14 days post‐injury, we detected high *SOX9* expression in cells surrounding the lesion site. Moreover, some of these cells were immunoreactive to the astroglial marker glial fibrillary acidic protein (GFAP) (Figure S1A, Supporting Information), suggesting that the microenvironment of contusive injury promotes the formation of *SOX9*‐ and GFAP‐expressing cells around the lesion core. To further evaluate the effects of the injured environment on the potency of transplanted hNSCs, we grafted GFP‐expressing hNSCs derived from human induced pluripotent stem cell (iPSC) line IMR90 into naïve rats (non‐injury) and SCI rats at 14 days post‐injury without matrigel‐embedded growth factors. After 2 months, the grafted cells scattered and remained viable, as evidenced by pronounced GFP expression (**Figure** **1**A). hNSCs grafted in non‐injury spinal cord showed substantial nerve fibers formation marked by MAP2^+^ with less *SOX9*+ cells and fewer GFAP+ astrocytes (Figure 1A, B; Figure S1B, Supporting Information). In contrast, hNSCs grafted into injured spinal cord tend to be organized into condensed clusters without axonal outgrowth across or beyond lesion sites (Figure 1A, B). Importantly, there were substantial numbers of GFP^+^ hNSCs expressing *SOX9* (49.4% ± 5.37% from injury versus 28.31± 3.91 from non‐injury) and GFAP^+^ astrocytes (41.11% ± 5.39% from injury versus 14.21± 2.64% from non‐injury) (Figure 1A,B; Figure S1B,C, Supporting Information). These results suggest that the injury environment induced and maintained *SOX9* expression in both endogenous NSCs and grafted hNSCs which tend to differentiate into astrocytes instead of neurons that may contribute to the limited tissue recovery. Apart from its key roles in maintaining NSC multipotency and glial specification,^[^ ^5^ ^]^ *SOX9* has been shown to function as a suppressor of neurogenesis in the CNS.^[^ ^5^, ^15^ ^]^ The genetic ablation of *SOX9* triggered early‐onset motor neuron differentiation in the developing mouse neural tube^[^ ^5a^ ^]^ and promoted locomotor recovery in SCI mice.^[^ ^7^ ^]^ This prompted us to investigate whether genetic manipulation of endogenous *SOX9* levels in hNSCs could induce the precocious acquisition of neuronal fate to enhance their therapeutic potential for treating SCI. We first examined the spatiotemporal expression of *SOX9* in three human pluripotent stem cell (hPSC) lines (embryonic stem cell (ESC) lines H9‐, Hes2‐, and iPSC line, IMR90)‐derived NSCs which were subjected to spinal motor neuron differentiation using a previous protocol (Figure 1C).^[^ ^16^ ^]^ We presented results using IMR90 cells in the rest of our studies as the other two ESC lines showed similar findings (Figure S1D, Supporting Information). We found the majority of cells were undifferentiated SOX2^+^ hNSCs with high levels of *SOX9*, whereas a few SOX2^−^ cells with low levels of *SOX9* had acquired neuronal identity with pan‐neuronal marker HuC/D expression. After 7 days of differentiation, we detected more HuC/D^+^SOX2^−^ cells with lower levels of *SOX9*. On day 21, the undifferentiated hNSCs had the same level of *SOX9*, but *SOX9* was no longer detectable in cells expressing MAP2 and differentiating motor neuron marker ISLET1/2 (Figure 1D). We further verified the gradual reduction of *SOX9* protein and mRNA levels was associated with corresponding increases in the expression of NeuN (pan‐neuronal marker) and ISLET1/2 in hNSCs up to day 21 post‐neuronal differentiation (Figure 1E, F). These results demonstrated that the graded reduction of *SOX9* expression in hNSCs was highly associated with the initiation of neurogenesis.

### 2.2 Dose‐dependent Expression of *SOX9* in Regulating hNSCs Survival, Renewal, and Differentiation

**Figure 2:**
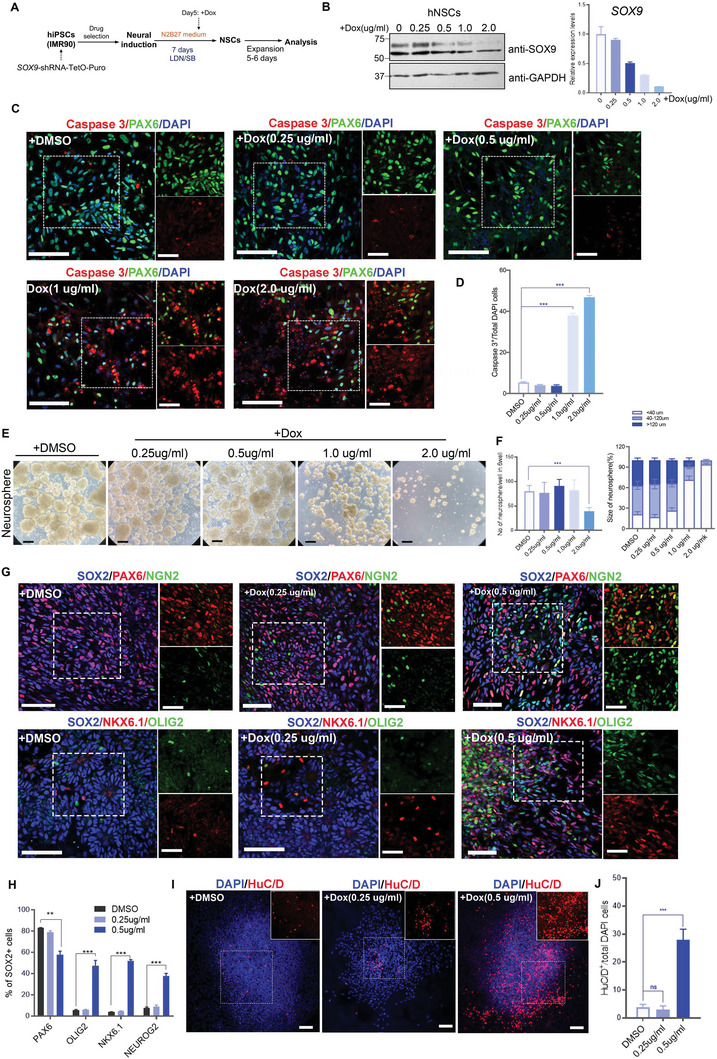
Dose‐dependent *SOX9* regulation of survival, renewal, and onset of differentiation in hNSCs. A) Experimental paradigm for B–H). (B) Dose‐dependent silencing of *SOX9* mRNA in hNSCs analyzed by immunoblotting and qPCR. C) Representative immunofluorescence images for Caspase 3, neural progenitor marker PAX6, and nuclei marker DAPI upon dose‐dependent inhibition of *SOX9* in hNSCs (Scale bar, 100 µm). White box shows a magnified view with indicated markers (Scale bar, 50 µm). D) Quantification of Caspase 3+ cells from (C). Student t‐test. E) Representative bright‐field images of neurospheres treated with DMOS as control and different dosages of Dox. F) Quantification of neurosphere number and size upon dose‐dependent inhibition of *SOX9*. One‐way ANOVA followed by Tukey's post‐hoc test. All data are expressed as mean ± SEM (Scale bar, 100 µm). Three independent experiments were performed. G) Representative immunofluorescence images for NEUROG2 (NGN2), SOX2, PAX6, NKX6.1, and OLIG2 upon dose‐dependent inhibition of *SOX9* in hNSCs (Scale bar, 100 µm). White box shows a magnified view with indicated markers (Scale bar, 50 µm) H) Percentage of PAX6^+^, OLIG2^+^, NKX6.1^+^, and NEUROG2^+^ in SOX2^+^ cells upon dose‐dependent inhibition of *SOX9*. One‐way ANOVA followed by Tukey's post‐hoc test. Three independent experiments were performed. I) Representative immunofluorescence images for HuC/D and DAPI in neurospheres upon dose‐dependent inhibition of *SOX9* (Scale bar, 100 µm). White box shows a magnified view with indicated markers. Three independent experiments were performed. J) Quantification of HuC/D^+^ cells from (I), Student t‐test. For all experiments, data are expressed as mean ± SEM, *p < 0.01, **p < 0.05, ***p <0. 005.

To further explore the link between graded reduction of *SOX9* levels and differentiation potential of hNSCs, we infected IMR‐90 with lentiviral‐mediated doxycycline (dox)‐inducible short hairpin RNA against the linker region of *SOX9* (*SOX9 KD1*) to bypass any undesirable cellular events during the pluripotent state and neural induction and examine the effects of graded reduction of *SOX9* expression on neurogenic potency of hNSCs at different dosages of dox (**Figure** **2**A). Following treatment with different doses of dox (0.5 to 2.0 µg mL^−1^) and DMSO as control 2 days before hNSCs formation, we observed *SOX9* mRNA and protein levels in hNSCs decreased in a dose‐dependent manner (Figure 2B). Examination of the viability of hNSCs by Caspase 3 immunostaining showed treatment with 1.0 or 2.0 µg mL^−1^ dox induced >50% reduction of *SOX9* expression in hNSCs but with extensive apoptosis, as evidenced by a marked increase in the number of Caspase 3^+^ cells. Treatment with 0.25 and 0.5 µg mL^−1^ dox resulted in ≈20% and ≈50% reduction of *SOX9* mRNA level, respectively, with moderately low levels of cell death that were comparable to the control (Figure 2B–D). We next examined whether reduced *SOX9* expression levels could affect the self‐renewal capacity of hNSCs using a neurosphere assay. We found that dox at 1.0 and 2.0 µg mL^−1^ (over 50% reduction of *SOX9*) resulted in the formation of smaller‐sized neurospheres compared to the lower dox concentrations (0.25 and 0.5 µg mL^−1^) and DMSO control (Figure 2E, F). In addition, only a few neurospheres were observed at 2.0 µg mL^−1^ dox due to extensive cell death (Figure 2D–F). These data imply that a moderate reduction of *SOX9* does not affect the survival and renewal capacity of hNSCs. We next examined the impact of reduced *SOX9* expression on the molecular properties of viable hNSCs treated with dox at 0.25 and 0.5 µg mL^−1^ and DMSO control. Within the SOX2^+^ hNSC population, we detected a reduction in the percentage of cells expressing PAX6 (57.9% ± 3.1%), which marks neural progenitors residing in the ventral to medial region of the neural tube, after treatment with 0.5 µg mL^−1^ dox compared to 0.25 µg mL^−1^ dox (79.1% ± 1.1%) and DMSO control (83% ± 0.23%) (Figure 2G, H). The reduced expression of PAX6 was accompanied by an increase in the proportion of cells expressing pro‐neural marker NEUROGENIN2 (NEUROG2) (37.5% ± 2.5%), ventral neural progenitor marker NKX6.1 (52.0% ± 1.1%), motor neuron progenitor marker OLIG2 (47.1% ± 1.0%), and HuC/D (27.9% ± 3.5%), whereas expression of these markers remained low in hNSCs treated with 0.25 µg mL^−1^ dox or DMSO control (Figure 2G–J), suggesting that ≈50% reduction of *SOX9* expression in hNSCs leads to early onset of neuronal differentiation which favors motor neuron lineage. Together, these findings indicate a dose‐dependent role of *SOX9* in regulating the onset of neurogenesis, self‐renewal, and survival of hNSCs.

### 2.3 Molecular Profiling and Characterization of Stable ≈50% Knockdown of *SOX9* hNSCs

**Figure 3:**
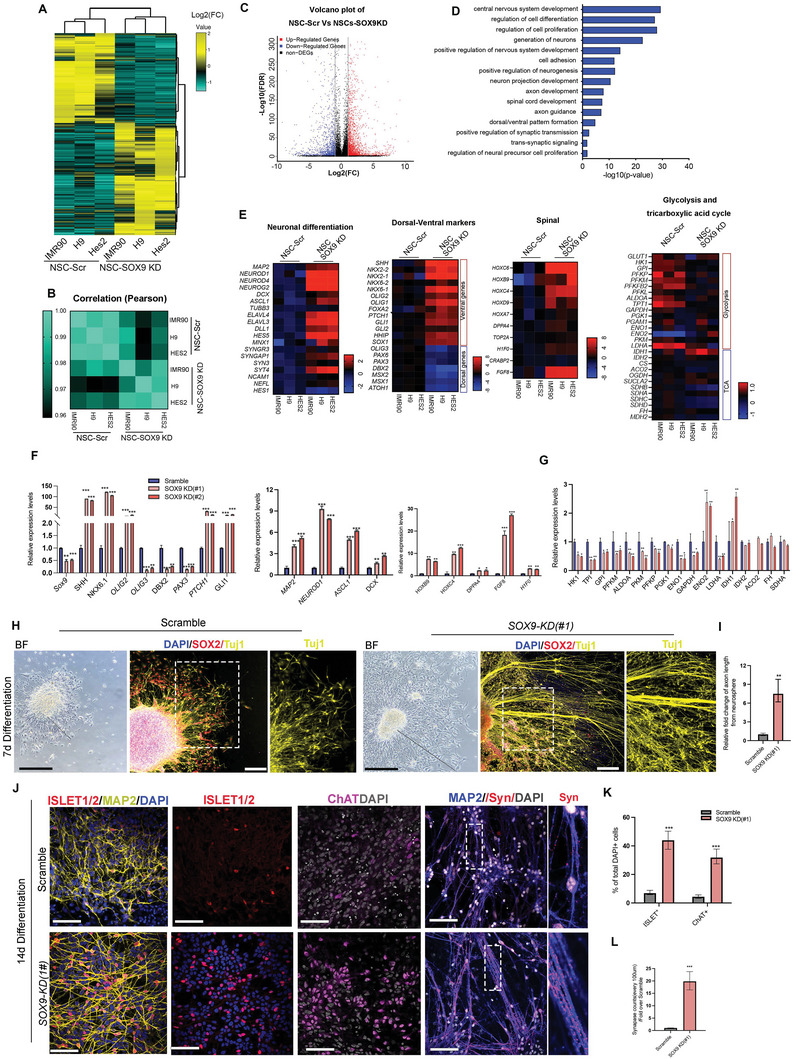
Moderate reduction of *SOX9* in hNSCs promotes robust neuronal differentiation and maturation. A) Heatmap shows the differential gene expression changes in the scramble and *SOX9 KD* hNSCs. B) The Pearson correlation coefficient between scramble and *SOX9 KD* hNSCs. (C) Volcano plot showing the differential levels of gene expression between scramble and *SOX9 KD* hNSCs. Red denotes significantly upregulated genes and blue denotes significantly downregulated genes. D) GO term analysis of upregulated genes from the RNA‐seq analysis of *SOX9 KD* hNSCs compared to scramble hNSCs. List of the top 15 GO terms ranked by *p*‐value (Fisher's exact test with Benjamini‐Hochberg correction). E) Heatmaps depict transcriptional changes in neuronal differentiation genes, dorsal‐ventral patterning genes, spinal genes, and key metabolic genes in glycolysis and tricarboxylic acid cycle (TCA) pathways in the scramble and *SOX9 KD* hNSCs. Red denotes upregulated genes and blue denotes downregulated genes. F) qPCR analysis of markers associated with neural tube patterning, neuronal differentiation, and spinal markers. One‐way ANOVA followed by Tukey's post‐hoc test. G) qPCR analysis of key metabolic genes in glycolysis and TCA pathways in the scramble and *SOX9 KD* hNSCs. H) Representative bright field (BF) images of scramble and *SOX9 KD* hNSCs and immunofluorescence for SOX2, TUJ1, and DAPI at 7 days differentiation in the absence of neurotrophic factors. Black line indicates the length of extended axons from the border of the neurosphere (Scale bar, 100 µm). White box shows the magnified view with indicated markers. I) Quantification of the relative length of extended axon in the scramble and *SOX9 KD* neurospheres. (*n* = 9 per group from three independent experiments). J) Representative immunofluorescence images for MAP2, ISLET1/2, ChAT, and synaptophysin (SYN) of scramble and *SOX9 KD* hNSCs at 14 days differentiation. White box shows a magnified view with indicated markers (Scale bar, 100 µm). K) Percentage of ISLET1/2‐ and ChAT‐positive cells in the scramble and *SOX9 KD* hNSCs after 14 days differentiation. Three independent experiments L) Quantification of synaptophysin counts between *SOX9 KD* and scramble at 14 days differentiation. Three independent experiments. Student's t‐test. For all experiments, data are expressed as mean ± SEM. *p < 0.01, **p < 0.05, ***p <0.005 versus scramble. Three independent experiments.

Previous data using dox inducible knockdown indicated that half‐dose *SOX9* expression in hNSCs exhibited distinct differentiation capacity and cell fate determination. To unravel the molecular profiling and regulatory signaling in hNSCs with half *SOX9* gene dosage, we used constitutive lentiviral vector encoding *shSOX9* and GFP to stably generate ≈50% *SOX9 KD1* (hereafter referred to as *SOX9 KD*) hNSCs derived from the three hPSCs lines (at passage 3 prior to the initiation of neuronal differentiation) for RNA‐sequencing (RNA‐seq) to identify differential gene expressions compared to the scramble control (Figure S2A, Supporting Information; Dataset 1). Hierarchical clustering revealed a significant proportion of commonly downregulated and upregulated genes with a good correlation of their expression levels between the three *SOX9 KD* cell lines (**Figure** **3**A–C). Gene ontology (GO) analysis of the differentially regulated genes showed highly enriched genes involved in the CNS development, regulation of cell proliferation, differentiation, and generation of neurons, among others (Figure 3D,E). Besides *SOX9 KD1*, we used an additional *SOX9* shRNA oligo (*KD2*) which targets *SOX9 5’ UTR* to validate the molecular changes detected in RNA‐seq by qPCR. Both *SOX9 KD* showed ≈50% reduction of *SOX9* expression in hNSCs (Figure 3F). Although the percentage of SOX2^+^ hNSCs in both *SOX9 KD* was similar to scramble control from passages (P) 1 to 3 when cultured in a low attachment plate supplement with neurosphere medium, their percentage gradually reduced from P4 onward due to the rapid onset of neuronal differentiation (Figure S2B, Supporting Information). Thus, molecular analysis, RNA‐seq, and grafting assay using P3 hNSCs indicate the transition from hNSCs to the pre‐neurogenic stage which confers their ability to undergo enhanced neurogenesis in vitro and in vivo. We, therefore, presented the qPCR validation results using IMR90‐derived hNSCs at P3 as the representative (Figure 3F‐L) and the other two cell lines showed similar observations (Figure S2C–H, Supporting Information). Among the differentially expressed target genes, *HOX* gene transcripts marking brachial (*HOXC6*), thoracic regions (*HOXB9* and *HOXC4*) of the neural tube, and FGF8 that determine spinal cord fate^[^ ^17^ ^]^ were upregulated in both *SOX9 KD*, whereas the expression of the pluripotent markers (*DPPA4* and *TOP2A*) were downregulated, suggesting that *SOX9 KD* had acquired brachial‐to‐thoracic spinal cord identity, which was further evidenced by the increased expression of spinal neural progenitor marker H1 histone family member 0 (*H1F0*) (Figure 3E, F). Additionally, *SOX9 KD* exhibited elevated expression levels of sonic hedgehog (*SHH*), and its signaling effector (*GLI1*), downstream target (*PTCH1*), and negative regulator (*HHIP*). Consistently, *SOX9 KD* showed markedly upregulated expression of floor plate marker *FOXA2* and ventral neural tube progenitor makers (*NKX2.2*, *NKX6.1*, *NKX6.2*, *OLIG1*, and *OLIG2*) in response to high levels of SHH signaling together with the corresponding motor neuron marker (*MNX1*) (Figure 3E, F). In contrast, the expression of medial‐to‐dorsal neural tube progenitors (*OLIG3*, *DBX2*, and *PAX3*) was downregulated (Figure 3E, F). Notably, we also detected robust elevated expressions of pro‐neural and pan‐neuronal markers in *SOX9 KD* (*MAP2, DCX, ASCL1, NEUROD1*, and *TUBB3*), and reduced expression of glycolytic genes (Figure. 3E, G).^[^ ^10^ ^]^

Cell intrinsic activation of SHH signaling in *SOX9 KD* hNSCs prompted us to examine whether they exhibit similar actions to SHH in promoting axonal projections/outgrowth, synapse formation, and motor neuronal maturation^[^ ^18^ ^]^ under the culture conditions without growth factor supplements to mimic the post‐injury environment. Similar‐size neurospheres (passage 3) derived from scramble control and *SOX9 KD* cells were cultured in Matrigel‐coated plates in the absence of neurotrophic factors (GDNF, BDNF, and IGF). After 7 days of culture, we observed robust axonal outgrowth, as labeled by pan‐neuronal marker TuJ1 in *SOX9 KD* cells, which exhibited thicker nerve fibers with much longer extension from the core of neurosphere compared to the scramble control (Figure 3H,I). Among the MAP2^+^ pan‐neuronal population, a substantial portion of the *SOX9 KD* cells expressed ISLET1/2 (43.9% ± 6.4%) and mature motor neuron marker choline acetyltransferase (ChAT) (31.8% ± 5.9%) that were associated with increased expression of presynaptic marker synaptophysin (Syn) (19.8% ± 3.4%) compared to the scramble control (2% ± 0.4%) (Figure 3J–L). Moreover, *SOX9 KD* neuronal cells showed increased action potential firing compared to the scramble control, as measured by current clamp recordings (Figure S3A,B, Supporting Information). These findings suggest that *SOX9 KD* hNSCs exhibit precocious neuronal differentiation biase toward motor neuron fate.

To demonstrate the specificity of *SOX9 KD*, we conducted lentiviral‐mediated overexpression of full‐length *SOX9* cDNA in *SOX9 KD*2 cells that resulted in reduced expression of *SHH*, its signaling effectors, and downstream targets together with *FGF8*, pan‐neuronal and pro‐neural markers to the levels that were comparable to that of scramble control (Figure S3C, Supporting Information). Consistently, the degree of axonal outgrowth, the portion of MN population, and its associated synapses were markedly reduced in the rescue treatment group (Figure S3D–G, Supporting Information). These results demonstrate the specificity of *SOX9 KD*, ruling out the possibility that alteration of gene expression is due to random integration of the viral construct into the cell genome.

### 2.4 Enhanced Neurogenic Potency of *SOX9* KD hNSCs is Partly Attributed to Reduced Glycolysis

Previous studies demonstrated that neuronal differentiation requires reduced glycolysis from NSCs whereas constitutive activation of glycolysis results in robust formation of astrocytes and neuronal death.^[^ ^10^ ^]^ Our RNA‐seq data and qPCR validation have confirmed a dramatic downregulation of glycolytic genes including *HK1*, *TPl*, *GPl*, *PFKM*, *LDHA*, *ALDOA*, *PKM*, *PFKP*, *ENO1*, and *GAPDH* in *SOX9 KD* hNSCs compared to the scramble control (Figure 3E,G). One exception was neuron‐specific enolase 2 (ENO2), which was upregulated by >2‐ and by 3 to 4‐fold in *SOX9 KD* hNSCs and their derived neurons, respectively (Figure 3E,G). In contrast to the general reduction of glycolytic genes expression in *SOX9 KD* cells, TCA genes remained largely unchanged, whereas isocitrate dehydrogenase 1 (IDH1), the mitochondria isoform of IDH, was elevated in *SOX9 KD* hNSCs and slightly increased in derived neurons (Figure 3E,G). These data imply that the enhanced neurogenic potency of *SOX9 KD* hNSCs may be attributed to their low glycolytic activity.

**Figure 4:**
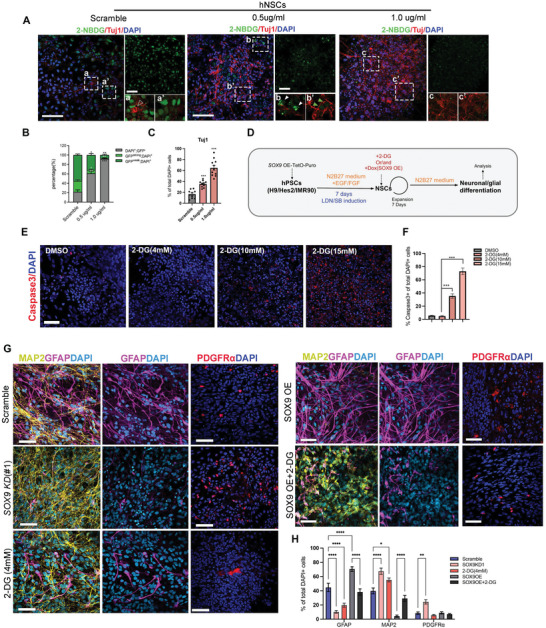
Reduced level of *SOX9* inhibits glycolysis in hNSCs. A) Representative images of immunolabeled TUJ1 (red) and fluorescent glucose (2‐NBDG) uptake in hNSCs with the scramble and dose‐dependent inhibition of *SOX9* expression. a‐a’, b‐b’, and c‐c’ show higher magnification with indicated markers. Empty arrows indicate 2‐NBDG‐negative cells with Tuj1 expression and white arrowheads indicate strong 2‐NBDG‐positive cells without Tuj1 expression. Green arrowheads indicate weak 2‐NBDG‐expressing cells. B) Quantification of different intensities of 2‐NBDG positive cells in (A). C) Quantification of Tuj1 positive cells in (A). D) Schematic diagram showing experimental strategy for *SOX9* overexpression (OE) and generation of neuronal/glial from different treatment groups. E) Representative immunofluorescence images for Caspase 3 upon dose‐dependent inhibition of glycolysis by 2‐DG inhibitor (Scale bar = 100 µm). F) Quantification of Caspase 3^+^ cells in (E). One‐way ANOVA followed by Tukey's post‐hoc test. G) Representative immunofluorescence images for MAP2, GFAP, and PDGFR‐*α* after 3 weeks differentiation of hNSCs with the indicated treatments (Scale bar = 50 µm). H) Quantification of GFAP+, MAP+, and PDGFR‐*α*+ cells in different treatments. One‐way ANOVA followed by Tukey's post‐hoc test. All data are expressed as mean ± SEM. For the above analysis, three independent experiments were performed. *p < 0.05, **p < 0.01, ****p < 0.0001.

To examine whether glycolytic activity in hNSCs is responsive to the alteration of *SOX9* expression levels, we treated cells with the fluorescent glucose tracer 2‐NBDG to measure the degree of glucose uptake.^[^ ^19^ ^]^ In agreement with the high glycolytic requirement of hNSCs,^[^ ^20^ ^]^ we detected abundant glucose uptake in scramble hNSCs, while a few differentiated TUJ1^+^ neurons were negative for 2‐NBDG. GFP signal of 2‐NBDG was considerably lower in ≈50% *SOX9 KD* (0.5ug uL^−1^ dox) hNSCs and barely detected in hNSCs with over 80% reduction of *SOX9* expression (1.0ug uL^−1^ dox), suggesting glycolytic activity in hNSCs is negatively correlated with *SOX9* expression level and neurogenesis (**Figure** **4**A,B). Despite more TUJ1+ cells generated from hNSCs with low *SOX9* (1.0ug uL^−1^ dox), we observed massive condensed nuclei stained with DAPI indicating severe apoptosis consistent with the previous observation (Figures 4A–C and 2C,D). We then determined whether reduced glycolytic activity contributes to *SOX9 KD*‐mediated robust neurogenesis by treating hNSCs with a competitive inhibitor of glycolysis, 2‐deoxy‐D‐glucose (2‐DG)^[^ ^21^ ^]^ at different concentrations (4, 10, and 15 mM) (Figure 4D). We found excess inhibition of glycolysis by 10 mM and 15 mM 2‐DG resulted in a graded increase in Caspase 3+ apoptotic cells compared to the viable cells treated with 4 mM 2‐DG and DMSO control, suggesting that low glycolysis is incompatible with the survival of hNSCs (Figure 4E,F). To evaluate the neuronal/glial differentiation capacity, hNSCs with different treatment groups were cultured in matrigel‐coated plates without neurotrophic factors (GDNF, BDNF, and IGF). After 21 days, GFAP+ astrocyte were more frequently observed in the scramble hNSCs (44.63±5.93%) whereas *SOX9 KD*‐ and 2‐DG‐ (4 mM) treated hNSCs differentiated predominantly into MAP2^+^ neurons (*SOX9 KD*:67.48±4.12%; 2DG: 55.21±2.79%; scramble: 39.75±5.94%) with a few GFAP^+^ astrocytes (*SOX9 KD*: 10.28±2.02%; 2‐DG: 19.69±2.88%) (Figure 4G,H). An injured environment promotes *SOX9* expression that eventually facilities astrocytes formation and inhibits neurogenesis.^[^ ^22^ ^]^ Consistently, *SOX9* overexpression (*SOX9* OE) in hNSCs resulted in the robust formation of GFAP+ astrocytes (*SOX9* OE: 70.55 ± 3.27%) with few neuronal cells. Glycolytic inhibition with 2‐DG could partially restore neurogenesis in *SOX9* OE cells by increasing MAP2+ neuronal population (*SOX9* OE + 2DG: 29.1±4.25%; *SOX9* OE: 4.26±1.54%) and reducing the number of GFAP+ astrocytes (*SOX9* OE + 2DG: 37.4±4.05%) (Figure 4G,H). In addition, *SOX9 KD* hNSCs showed a significant increase in oligodendrocyte population expressing PDGFR*α* compared to scramble (*SOX9 KD*: 24.34 ± 3.02%; scramble: 8.45±1.75%) (Figure 4G,H). No significant difference in the number of PDGFR‐positive cells was observed between scramble, 2‐DG treatment, *SOX9* OE, and *SOX9* OE +2‐DG groups (Figure 4G,H). Together, these results suggest that the enhanced neurogenic potency of *SOX9 KD* hNSCs is partly attributed to the inhibition of glycolysis.

### 2.5 *SOX9 KD* hNSCs Inhibit CSPG Expression and Promote the Restoration of Motor‐sensory Neural Circuitry

**Figure 5:**
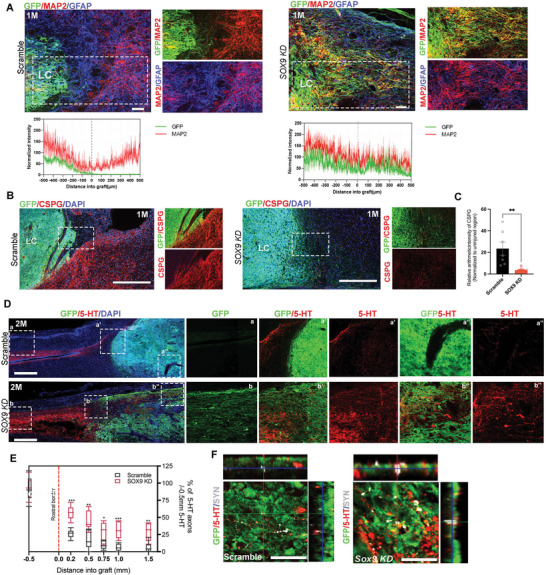
*SOX9 KD* grafts exhibit efficient distribution into lesion cavities and integration in the absence of growth factors in the SCI model. A) Representative immunofluorescence images for GFP, MAP2 (red), and GFAP (Blue) in sagittal sections with scramble and *SOX9 KD* grafts at 1 month (1 m) post‐graft. The cystic lesion cavity (LC) is surrounded by dense GFAP immunoreactivity (blue) in scramble control, whereas GFP^+^ *SOX9 KD* grafts extend across GFAP barriers. White box shows a magnified view with indicated markers (Scale bar, 100 µm). Lower panel shows the line scan of fluorescence intensity with indicated markers across the lesion cavity of recipients with scramble and *SOX9 KD* grafts. (*n* = 7 rats per group, 4–5 sections per rat) B) Representative immunofluorescence images for GFP, CSPG (red), and DAPI (Blue) in spinal cord sagittal sections grafted with the scramble and *SOX9 KD* hNSCs at 1‐month post‐graft. The cystic lesion cavity (LC) formed with surrounding dense CSPG immunoreactivity (red). White box shows a zoomed‐in view with indicated markers. *SOX9 KD* grafts attenuated CSPG graft/host interface (Scale bar, 500 µm). C) Relative fluorescence intensity analysis of CSPG surrounding the lesion cavity that normalized to the uninjured region (*n* = 7 rats per group, 4–5 sections per rat). **p < 0.05. D) Representative images of host serotonergic axons immunolabeled with 5‐HT (red) extending into the scramble and *SOX9 KD* grafts at 2 months post‐transplantation. a‐a’’ and b‐b’’ show high magnification of 5‐HT^+^ fibers innervating into the graft at different regions from rostral to caudal (Scale bar, 100 µm). E) Quantification of the portion of host 5‐HT^+^ serotonergic axons in the scramble and *SOX9 KD* grafts, normalized to the total number of 5‐HT^+^ axons located 0.5 mm rostral to the lesion site (*n* = 6 rats per group, 3–4 sections per rat). One‐way ANOVA. **p < 0.05, ***p < 0.005. F) Triple immunolabeling of host 5‐HT^+^ fibers, proximal hsyn, and GFP‐terminals indicating the establishment of synaptic contacts between the host raphespinal fibers and *SOX9 KD* grafted cells (*n* = 6 rats per group, 3–4 sections per rat, Scale bar, 50 µm).

**Figure 6:**
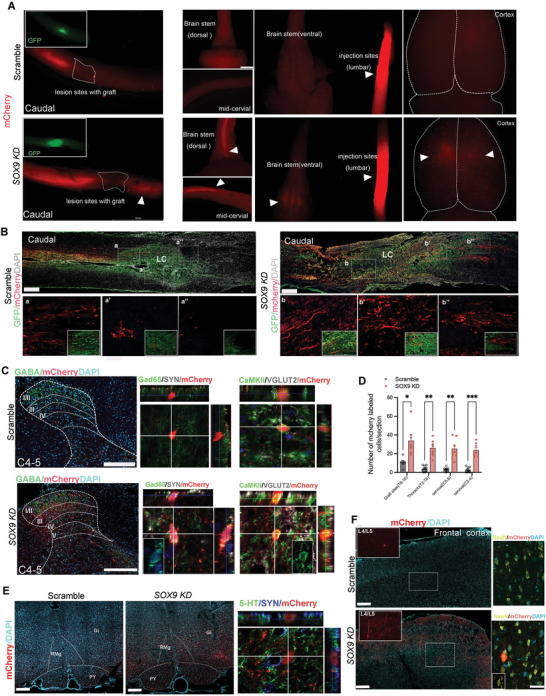
Retrograde tracing of host connectivity with neurotropic viruses. A) Macroscopical view of retrograde labeling of host projection following injections of AAV2‐retro‐Syn‐mCherry in the lumbar spinal cords at 3 months post‐graft. The left panel shows mCherry labeling of host connectivity in the T8 injured spinal cord grafted with the scramble and *SOX9 KD* hNSCs. Insets show grafts expressing GFP. White arrowheads indicate traced host mCherry‐expressing cells rostral to the *SOX9 KD* graft in the lesion site. The right panel shows strong expression of mCherry in the lumbar (L2) injection sites and retrogradely traced host mCherry‐expressing cells in the cervical spinal cord, brainstem (ventral and dorsal), and cortex rostral to the T8 SCI site. Scale bar, 500 µm. B) Sagittal section showing retrograde labeling of host cells expressing mCherry from caudal to rostral in injured spinal cords with the scramble and *SOX9 KD* grafts at 3 months post‐transplantation. High magnification of mCherry^+^ projections along the grafts from rostral to caudal is shown in panels a‐a’’ and b‐b’’. Numerous mCherry^+^ host neurons are scattered throughout the *SOX9 KD* graft. Scale bar, 200 µm. C) Retrograde labeling host cells at the cervical level (C4‐5) in lamina I‐IV, with inner lamina I/II indicated by GABA (green) staining. Triple labeling for mCherry/SYN/Gad65 and mCherry/VGLUT2/CaMKII at the cervical spinal cord level. Scale bars, 100 µm. D) Quantification of the number of mCherry^+^ cells on cross‐sections of injured spinal cords with the scramble and *SOX9 KD* grafts at thoracic and cervical levels. E) Retrograde mCherry+ projections were founded in the caudal raphe magnus nucleus (RMg), gigantocellular reticular nucleus (*G*i), and pyramidal tract (PY) within the brainstem of recipients with *SOX9 KD* grafts but not with scramble grafts. The panel shows projected mCherry‐expressing cells forming synaptic connections with 5‐HT+ serotonergic neurons in RMg. Scale bar, 500 µm. F) Retrograde mCherry+ projections were found in the somatosensory cortex of recipients with *SOX9 KD* grafts and co‐localized with neurons (NeuN, yellow), which display a classical pyramidal cell morphology. Scale bars, 500 µm. Student t‐test. All data are presented as mean ± SEM. *n* =  7‐8 rats per group, at least five sections per rat were analyzed for immunohistochemistry. *p < 0.05, **p < 0.01,***p < 0.001.

We examined whether *SOX9 KD* hNSCs exhibited enhanced therapeutic value in treating severe SCI by using a clinically relevant rat model of thoracic contusion injury. As expected, a lesion control without grafts showed the formation of cystic cavitation surrounded by a glial scar expressing both GFAP and CSPG, which prevents axons and corticospinal tract fibers (CST) from penetrating through the lesion cavity that disrupts neuronal connectivity (Figure S4A,B, Supporting Information). A total of 2 × 10^5^ GFP‐expressing scramble control or *SOX9 KD* hNSCs were grafted into the lesion site (1 µL administered into the left and right sides of the injury site) without growth factors supplementation at 14 days post‐injury. Anatomical analysis showed both scramble and *SOX9 KD* hNSCs could expand and survive in grafted animals from 1 to 3 months post‐graft (Figure S5A, Supporting Information), suggesting successful integration of host tissues in the lesion site to prevent further tissue loss. There was no sign of tumor formation and unlimited cell expansion in both treatments as evidenced by regional restrictions of GFP intensity and the absence of KLF4 expression which is one of the iPSC reprogramming factors that could trigger tumor formation upon transplantation of iPSCs (Figure S5B–E, Supporting Information). We observed that grafted *SOX9 KD* hNSCs were more uniformly dispersed and completely filled the lesion cavity, whereas control grafts tend to form compact clusters, as shown by an irregular distribution of GFP^+^ cells (Figure S5A, Supporting Information). Consistent with our in vitro study, longitudinal sections revealed that grafted *SOX9 KD* cells generated substantial amounts of MAP2^+^ neuronal cells with massive axonal branches that penetrated the GFAP‐expressing glial scar as early as a 1‐month post‐graft. Despite the occurrence of neuronal differentiation in grafted control cells, more graft cells expressed GFAP with fewer axonal projections across the glial scar (**Figure** **5**A). Following the SCI, CSPGs are produced by reactive astrocytes, which migrate to the lesion site to form the glial scar. The presence of CSPGs is considered to be a main inhibitory component that limits axonal outgrowth and regeneration, oligodendrocyte replacement, and remyelination.^[^ ^23^ ^]^ In agreement with the literature,^[^ ^24^ ^]^ upregulated CSPG expression formed a complex barrier around the lesion sites in response to the injury, restricting nerve regeneration and outgrowth of grafted control cells (Figure 5B,C; Figure S4A,B, Supporting Information). Surprisingly, we found that *SOX9 KD* graft could markedly reduce CSPG deposition around lesion sites that facilitate extension of GFP‐expressing axons across the glial scar for reconstituting neuronal connections (Figure 5A–C). This indicates the transplanted *SOX9 KD* hNSCs may exert non‐cell autonomous effects on the injured environment. Subsequently, we determined whether grafted cells could re‐establish the neuronal circuits across the lesion by analyzing descending pathways. SCI results in the disruption of serotonergic raphespinal projections to spinal motor areas, which is critical for the modulation of locomotion, muscle tone, and sensory responses.^[^ ^25^ ^]^ At 2 months post‐graft, we detected robust caudally descending 5‐HT^+^ serotonergic projections into the *SOX9 KD* graft at a long distance. In contrast, less dense and shorter 5‐HT^+^ axons penetrated into the control grafts consistent with previous transplantation studies which showed limited host axon penetration at 2 months post‐graft (Figure 5D,E).^[^ ^2^, ^26^ ^]^ Importantly, the 5‐HT labeled fibers regenerated into the *SOX9 KD* graft were colocalized with the human pre‐synaptic marker synaptophysin (SYN) as well as the GFP‐positive axonal projections, indicating the establishment of host‐graft synaptic connectivity (Figure 5F). In contrast, synaptic contacts between the host serotonergic raphespinal fibers and scramble grafts were barely detected (Figure 5F). To further consolidate the observations, we conducted retrograde tracing in the grafted animals using neurotrophic viruses AAV2‐Retro‐Syn‐mCherry to label the connectivity of neuronal circuitry from the spinal cord to the cortical region.^[^ ^27^ ^]^ Animals received bilateral injections of AAV2‐Retro‐Syn‐mCherry into the lumbar spinal cords 3 months after transplantation of scramble control hNSCs or *SOX9 KD* hNSCs. Macroscopical images showed mCherry^+^ cells can be visualized in lumbar region and caudal to the grafts in the thoracic, but not in the cervical spinal cord, brainstem, and cortex of animals receiving GFP^+^ scramble control (**Figure** **6**A,D). Detailed examination of the sagittal section from the grafted spinal cord showed the presence of a few mCherry^+^ cells in the GFP^+^ scramble control grafts (Figure 6B,D). In contrast, retrogradely labeled mCherry‐expressing cells can be visualized from the posterior to the anterior region of *SOX9 KD* grafts, in the cervical spinal cord, brainstem, and cortex (Figure 6A). Detailed examination of the sagittal section from the grafted spinal cord showed a large number of projected mCherry neurons are scattered throughout the *SOX9 KD* grafts to rostral regions where only a few mCherry^+^ cells in the GFP^+^ scramble control grafts can be detected (Figure 6B,D). Consistently, a substantial number of projected mCherry^+^ cells can be visualized in the cervical spinal cord, brainstem, and cortical region in animals transplanted with *SOX9 KD* hNSCs which can be barely detected in scramble graft (Figure 6A–D), indicating reconstitution of damaged neural circuitry upon *SOX9 KD* grafts. Additionally, numerous mCherry^+^ cells were projected into interneuron subtypes associated with sensory functions such as Gad65 (for GABAergic inhibitor neurons), calcium‐calmodulin kinase 2 (CaMKII, for excitatory neurons), and vesicular glutamate transporter (VGLUT2, for excitatory neurons) in the dorsal horn of the cervical spinal cords from the *SOX9 KD* grafted animals, whereas scramble control grafts seldom had the mCherry^+^ cells projecting to these neuronal population (Figure 6C). Some of the mCherry^+^ Gad65^+^ neurons were co‐labeled with SYN, indicating the synaptic connection of host cells with GABAergic neurons (Figure 6C). The projection of mCherry^+^ cells into the brainstem of recipients with *SOX9 KD* hNSCs formed a synaptic connection with 5‐HT^+^ in the raphe magnus nucleus (Figure 6E) which further confirms our findings of direct connection of host serotonergic axons with spinal *SOX9 KD* grafts (Figure 5D–F). A similar degree of projection from mCherry‐expressing cells was found in NeuN^+^ neurons which display a classical pyramidal morphology in layers 4/5 of the somatosensory cortex from animals receiving *SOX9 KD* grafts (Figure 6F). There were no mCherry^+^ cells detected in the frontal cortex from animals with scramble control grafts (Figure 6F).

We also conducted intracortical injections of biotinylated dextran amine (BDA) for anterograde tracing of corticospinal tract (CST), another descending pathway from the cerebral cortex to the spinal cord that is involved in controlling voluntary movement in humans.^[^ ^28^ ^]^ Consistent with previous human stem cell implantation studies,^[^ ^2^, ^26^ ^]^ most BDA‐labeled corticospinal axons regenerated into the scramble hNSC grafts at 0.5 mm from the rostral border with some extending up to a distance of 2 mm (Figure S6A,B should be S6A,C Supporting Information). In *SOX9 KD* hNSC grafts, CST exhibited more extensive penetration with a substantial proportion of axons at 2.5 mm when normalized to the total number of CST axons located 0.5 mm rostral to the lesion site (Figure S6A,B, should be S6B, C Supporting Information). These results indicate that the grafted *SOX9 KD* hNSCs are more effective in re‐establishing connectivity with host neurons involved in motor and sensory functions, with subsequent growth of long‐distance axonal projections in the traumatic SCI.

### 2.6 Robust Neuronal Differentiation and Maturation Potency of *SOX9 KD* Grafts

**Figure 7:**
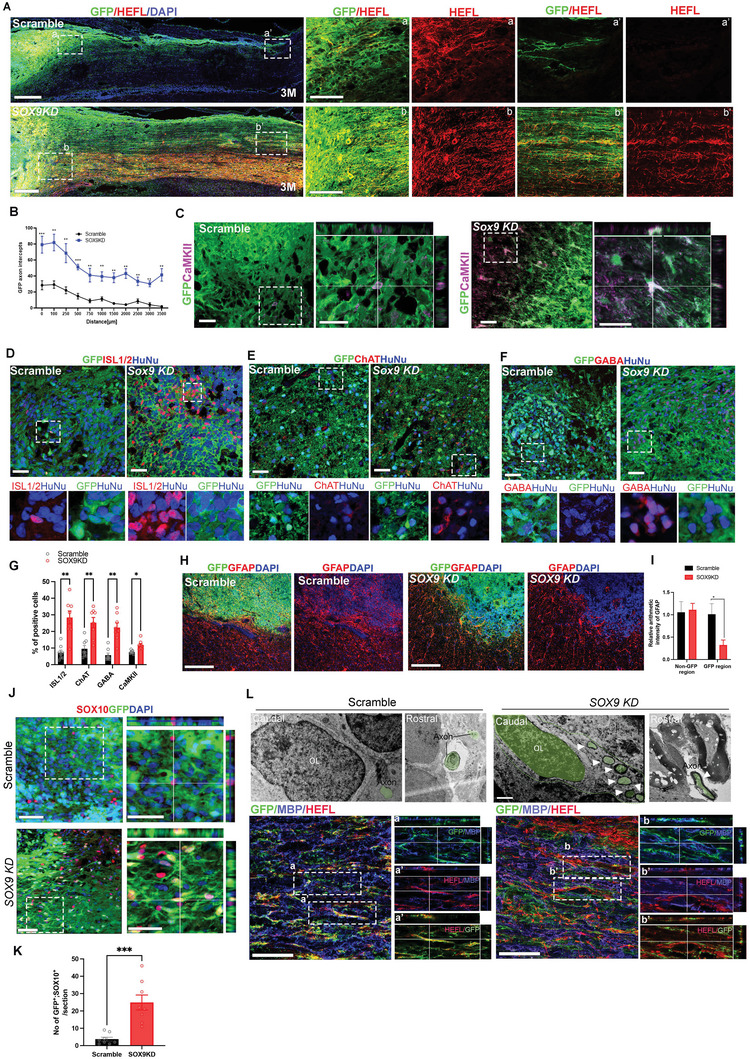
*SOX9 KD* hNSC grafts display enhanced differentiation capacity toward beneficial neuronal subtypes in the absence of growth factors in the SCI model. A) GFP and HNEFL immunolabeling in spinal cord sagittal sections revealed GFP‐expressing *SOX9 KD* grafts at injured sites generated robust axons extending into the host spinal cord caudally after 3 months post‐graft. a‐a’’ and b‐b’’ indicate higher magnification of HEFL‐positive fibers in the graft at different regions of rostral to caudal (*n* = 6 rats per group, 3–4 sections per rat, Scale bar, 100 µm). B) Quantification of axon intercepts at specific distances from graft‐host border in the injured cord grafted with scramble and *SOX9 KD* hNSCs. **p < 0.01, ***p < 0.001, one‐way ANOVA with Bonferroni. C) Grafts from scramble and *Sox9 KD* immunolabeled with GFP and excitatory neurons (CaMKII), and D) HuNu antibody to confirm the human origin of the cells together with differentiating motor neuronal marker (ISL1/2), E) mature motor neuronal marker choline acetyltransferase (ChAT), and F) inhibitory neuronal marker (GABA) at 2 months post‐graft. White box shows a zoomed‐in view of the co‐localization of the indicated markers (Scale bar = 50 µm). G) Quantification of the percentage of neuronal subtypes (ISL1/2, ChAT, GABA, and CaMKII) among HuNu‐positive or GFP‐positive scramble and *SOX9 KD* grafts (*n* = 7 rats per group, 3–4 sections/rat). *p < 0.05, **p < 0.01, **p < 0.005. H) GFP‐positive grafts from the scramble and *SOX9 KD* were immunolabeled with astrocytes marker (GFAP, red) 3 months post‐graft. Dashed lines indicate graft/host border (Scale bar = 200 µm). I) Quantification of GFAP relative intensity in graft/non‐graft regions of the injured cord grafted with the scramble and *SOX9 KD* grafts (*n* = 7–8 rats per group, 3–4 sections/rat). J) GFP‐positive grafts from the scramble and *SOX9 KD* immunolabeled with oligodendrocyte marker (SOX10, red) at 3 months post‐graft. White box shows a zoomed‐in view of the co‐localization of indicated markers (Scale bar = 50 µm). K) Quantification of the number of SOX10‐ and GFP‐positive cells in the injured cord grafted with scramble and *SOX9 KD* hNSCs (*n* = 7–8 rats per group, 3–4 sections/rat). ***p < 0.005. L) Transmission electron microscopy showed that diaminobenzidine (DAB)‐labeled GFP‐expressing scramble and *SOX9 KD* grafts in the injury sites and caudal region. GFP^+^ cells deposited with DAB in *SOX9 KD* grafts can form oligodendrocytes (OL) and human axons that are wrapped by myelin‐like structures (white arrowheads). Scale bars, 1 mm. Lower panels show GFP‐positive grafts from scramble and *SOX9 KD* immunolabeled with HNEFL(red) and myelination marker (MBP, blue) at 3 months post‐graft. Insets of a‐a’ and b‐b'show the co‐localizations of GFP‐positive grafts with indicated markers in the injury site (Scale bar, 100 µm). *n* = 6 rats per group for EM. *n* = 8 rats per group for fluorescence immunostaining. All data are presented as mean ± SEM.

To further determine whether grafted IMR90‐derived *SOX9 KD* hNSCs can surmount the injury environment with a lack of growth factors and generate therapeutic cell types, we spatiotemporally examined the regenerative properties of the scramble and *SOX9 KD* hNSCs. Consistent with in vitro data, we detected a significant increase in the number of TUJ1^+^ neuronal populations derived from *SOX9 KD* grafts as early as 2 weeks post‐graft, which progressively increased up to 4 weeks compared to the control grafts, indicating early onset of neurogenesis in *SOX9 KD* grafts (Figure S7A,B, Supporting Information). As expected, SOX9 expression remained high in the control grafts and fewer HNEFL+ neurons (axonal marker human‐specific neurofilament) were formed at 4 weeks post‐graft in the injured host spinal cord (41.35% ± 2.96% of GFP+ cells expressed SOX9, 34.89% ± 5.6% of cells had the mean intensity of HNEFL) (Figure S7C,D, Supporting Information). On the contrary, GFP+ cells from *SOX9 KD* grafts differentiated into more HNEFL^+^ neurons with a marked reduction of SOX9 expression (18.18% ± 2.86% of GFP+ cells expressed SOX9, 56.0% ± 7.0% of cells had mean intensity of HNEFL) (Figure S7C,D, Supporting Information). Importantly, we observed a large number of GFP^+^ axons co‐expressing HNEFL emerging from *SOX9 KD* grafts into the injured spinal cord and extending much further caudally by >35 mm at 3 months post‐graft (**Figure** **7**A,B) that recapitulated long nerve fibers extension in vitro (Figure 3H,I). Although the control grafts also showed GFP^+^ axonal outgrowth, they were relatively less in number with shorter extensions up to 25 mm caudally (Figure 7A,B). In addition, only a subset of GFP^+^ axons from control grafts expressed HNEFL (Figure 7A), suggesting some of the neurons were in a less mature stage.

We further examined specific neuronal subtypes derived from the grafts at 2 months post‐transplantation. In line with previous findings,^[^ ^2^, ^26^ ^]^ scramble grafts gave rise to relatively fewer motor neurons (ISL1/2, 6.44% ± 1.7%; ChAT, 9.96% ± 2.1%), excitatory neurons (CaMKII, 4.33% ± 0.82%), and inhibitory GABAergic neurons (GABA, 6.11% ± 1.7%) (Figure 7C–G) compared to moderate amounts at 6 months post‐graft in the presence of matrix enriched with growth factors as shown by previous studies.^[^ ^2^, ^26^ ^]^ Consistent with the in vitro observation, *SOX9 KD* grafts marked by human nuclear antigen (HuNu) favored motor neuron fates (ISL1/2, 29.2% ± 4.53%; ChAT, 26.7% ± 3.55%) (Figure 7D,G). In addition, we detected a moderate increase in the populations of CaMKII^+^ excitatory neurons (7.8% ± 0.98%) and GABA^+^ neurons (20.34% ± 4.6%) from *SOX9 KD* grafts (Figure 7C,E–G). Compared to control grafts, only a small proportion of grafted *SOX9 KD* cells differentiated into GFAP^+^ astrocytes at 3 months post‐graft (Figure 7H,I). A previous report showed that Sox10 could compensate for the loss of oligodendrocyte progenitors in *SOX9‐*deficient mouse NSCs.^[^ ^5a^ ^]^ Consistently, *SOX9 KD* grafts exhibited upregulated SOX10 expression in a cell‐autonomous manner, whereas it was barely detectable in the control grafts (Figure 7J, K). Consequently, *SOX9 KD* hNSCs not only differentiated into neurons but also oligodendrocytes which can myelinate their own axons, as evidenced by the juxtaposition of myelin basic protein (MBP)‐labeled myelin sheath and GFP^+^ HNEFL^+^ axons, whereas no MBP expression was detected along the axons emerging from the control graft from high‐resolution confocal imaging with Z‐stack (Figure 7L, lower panel). Our results demonstrated superiority of *SOX9 KD* grafts in generating myelinated human axons in vivo, which are found to be absent from prior studies in rodent hosts due to a lack of inter‐species recognition.^[^ ^2^, ^29^ ^]^ We then examined molecular changes that are associated with SHH signaling and metabolic pathways underlying differential adaptation, integration, and neurogenic potency of *SOX9 KD* grafts in the injured spinal cord. We performed fluorescence‐activated cell sorting (FACS) to isolate GFP^+^ transplanted cells from the injured spinal cord at 2 months post‐graft for gene expression analysis (Figure S7E, Supporting Information). Consistent with the in vitro findings, *SOX9 KD* grafts exhibited downregulated expression of glycolytic genes and upregulated ENO2 without altering TCA gene expression compared to the scramble control (Figure S7F, Supporting Information). In addition, sorted *SOX9 KD* cells showed elevated expression of pro‐neural markers, subtypes of neuronal markers, and SHH targets (Figure S7G, Supporting Information). Altogether, these results demonstrate the intrinsic superiority of *SOX9 KD* hNSCs for generating therapeutic neural cell types for the treatment of SCI without reliance on growth factor‐enriched matrices.

### 2.7 *SOX9 KD* Grafts Improve Hindlimb Function After Contusive SCI

**Figure 8:**
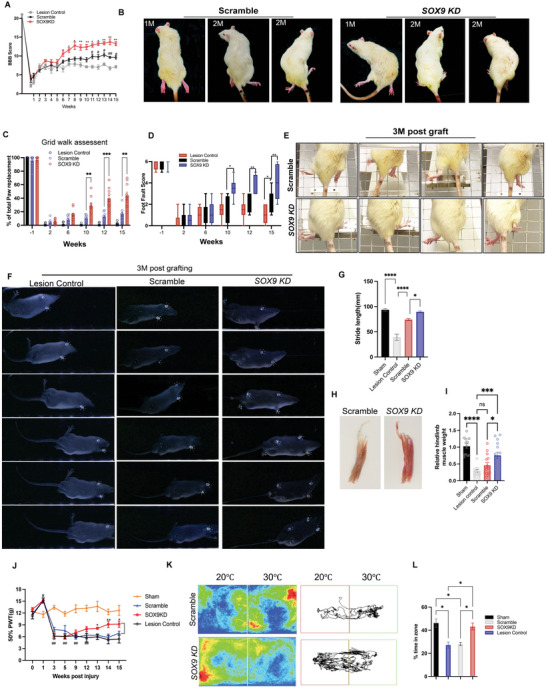
Significant functional improvement after transplantation of *SOX9 KD* hNSC grafts into contusive SCI. A) BBB scores of lesion control, and pre‐and post‐grafting with scramble and *SOX9 KD* hNSCs. Two‐way repeated‐measures ANOVA followed by post‐hoc Fisher's exact test. *p < 0.05, **p <0.01 *SOX9 KD* versus scramble; #*p* <0.05, ##*p* < 0.02 scramble versus lesion control. B) Representative images showing hind limb movement in grid walking of SCI rats grafted with scramble and *SOX9 KD* hNSCs at 1 and 2 months (M) post‐graft. C) Grid walk quantitative analysis measured as a percentage of hind limb placement. One‐way ANOVA with Tukey's post‐hoc test; *p < 0.05, **p < 0.01. D) Foot fault score analysis of hind limb measured by rating scale for foot placement in the skilled ladder rung walking test (correct placement = 6 points; partial placement = 5 points; placement correction = 4 points; replacement = 3 points; slight slip = 2 points; deep slip = 1 point; and total miss = 0 points). One‐way ANOVA with Tukey's post‐hoc test; *p < 0.05, **p < 0.01. E) Representative images showing foot placement during ladder rung walking in SCI rats grafted with scramble and *SOX9 KD* hNSCs after 3 months post‐graft. F) Representative video images showing footprints of SCI (lesion control) and SCI rats grafted with scramble and *SOX9 KD* hNSCs after 3 months post‐graft. G) Quantification of stride length in sham, SCI(lesion control), and SCI rats with scramble and *SOX9 KD* grafts. Student's t‐test. *p < 0.05, **p < 0.01. H) Representative images of hind limb muscle from SCI rats grafted with scramble and *SOX9 KD* hNSCs after 3 months post‐graft. I) Relative quantification of muscle weight from sham, SCI (lesion control), and SCI rats grafted with scramble and *SOX9 KD* NSCs. One‐way ANOVA followed by Tukey's post‐hoc test. *p < 0.05, ***p < 0.001, ****p < 0.0001. J) Time course of the mechanical allodynia, as measured by the von Frey force threshold for withdrawal in sham and SCI rats with scramble and *SOX9 KD* grafts. *p < 0.05, **p < 0.01 *SOX9* KD versus scramble; ##*p* <0.01 *SOX9 KD* versus sham. Two‐way repeated‐measures ANOVA. K) Representative track imaging of the two‐plate preference test at 30 °C versus 20 °C after 3 months post‐graft (14 weeks post‐injury). L) Occupancy quantification of 30 °C versus 20 °C after 3 months post‐graft. *p < 0.05; Two‐tailed unpaired Student's t‐test. All data are expressed as mean ± SEM. *n* = 9 (scramble); *n* = 8 (lesion Control); *n* = 10 (*SOX9 KD)*; *n* = 9 (Sham).

Grafted animals were subjected to motor function tests to determine whether *SOX9 KD* grafts can promote functional recovery after SCI compared to scramble grafts. Hindlimb locomotor activity in the lesion control, scramble hNSCs, and *SOX9 KD* hNSCs were assessed weekly by the Basso, Beattie, and Bresnahan (BBB) locomotor scale starting 7 days after injury. At 1 and 2 weeks post‐injury, all treatment groups showed substantial loss of locomotor function with an average BBB score < 5 (little or no hindlimb movement). Scramble recipients exhibited improved locomotor function from 12 weeks (10 weeks post‐graft) compared to lesion control whereas the *SOX9 KD* recipients began to show significant improvement in hindlimb motor function from 6 weeks (4 weeks post‐graft) to the end of the experiment (15 weeks post‐injury) compared to scramble recipients animals (**Figure** **8**A). In addition, the *SOX9 KD* recipients showed a remarkable improvement in plantar stepping with frequent weight support and hindlimb coordination from 8 weeks (2 months) post‐injury compared to scramble recipients (Figure 8B). To further evaluate the skilled locomotor function and coordination, we employed the grid‐walking test by counting the percentage of correct steps out of paw replacements and foot faults as rats traversed the metal grid.^[^ ^30^ ^]^ The *SOX9 KD* grafted rats exhibit much better performance in placing their affected hind paw (left and right) correctly on the grid with fewer misdirected steps from 10 weeks post‐injury compared to the scramble recipients whereas scramble recipients showed a mild improvement by weeks 15 compared to lesion control (Figure 8C–E; Figure S8A, Supporting Information). Subsequently, the gait of the transplanted animals was assessed by the footprint test, in which the pressure exerted by the feet during locomotor activity was converted into a digital image of the plantar surface by a force sensor, indicating limb stepping ability and coordination. As expected, the sham group showed clear paw position and toes movement (Figure 8F, Figure S8B) whereas the lesion control lost forelimb‐hindlimb coordination and showed a shuffling gait (Figure 8F). Although scramble recipients exhibited occasional forelimb‐hindlimb coordination, the *SOX9 KD* recipients showed much better recovery of paw positions and toe movement (Figure 8F). Quantitative analysis of stride length in grafted animals further confirmed a marked improvement in locomotion activity of *SOX9 KD*‐grafted animals with values higher than the scramble and lesion controls (Figure 8G). Accordingly, there was an increased hind limb muscle weight in the *SOX9 KD* group compared to the scramble and lesion control group, indicating recovery from muscle loss due to motor neuron damage following SCI (Figure 8H,I). Besides the debilitating sequalae from defective locomotion, SCI also leads to somatosensory impairment, resulting in hypersensitivity (e.g., central neuropathic pain) or hyposensitivity.^[^ ^31^ ^]^ We did not detect mechanical allodynia and hyperalgesia in the first 2 weeks post‐injury, but injured rats showed increased withdrawal responses to mechanical stimuli compared to baseline (Figure 8J), indicating the development of hyposensitivity, which could be due to “spinal shock” associated with the transient acute shutdown of sensorimotor function after SCI.^[^ ^32^ ^]^ Consistent with previous findings,^[^ ^31^, ^32^ ^]^ SCI rats from all treatments developed mechanical allodynia hyperalgesia at 3 weeks post‐injury compared to baseline and the sham group (Figure 8J). The *SOX9 KD* grafts significantly alleviated mechanical allodynia from 13 weeks post‐injury (11 weeks post‐graft compared to scramble grafts which showed no improvement in mechanical hypersensitivity (Figure 8J). Two temperature preference tests further confirmed that *SOX9 KD* grafts could modulate cold avoidance effects, indicating an improvement in cold hyperalgesia (Figure 8J–L; Figure S8C, Supporting Information). These findings demonstrate the enhanced therapeutic potential of *SOX9 KD* grafts for the restoration of locomotor and somatosensory function in a rodent model of contusion SCI.

## 3 Discussion

Traumatic spinal cord injury, commonly induced by compression, contusion, or laceration, can lead to severed axons and neuronal death, resulting in motor and somatosensory dysfunction. Consequently, SCI patients can experience permanent paralysis and/or varying degrees of impaired sensation depending on the level and severity of the injury. Failure of nerve tissue regeneration following SCI can be due to both intrinsic and extrinsic inhibitory factors in the post‐injury environment. Adult spinal cord neurons lose their intrinsic capacity for axonal regeneration after injury.^[^ ^33^ ^]^ Moreover, the ability of severed axon regrowth across the lesion to re‐establish connectivity with host neurons is further impeded by the inflammatory micro‐environment and condensed astrocytic scars around the lesion core.^[^ ^1^, ^3^, ^34^ ^]^ Transplantation therapy for SCI can provide an alternative strategy for restoring neural tissue, however, the functional outcome largely depends on the survival, differentiation potency, integration capacity, and axial identity of the grafted hNSCs.^[^ ^2^, ^35^ ^]^ Given the hostile micro‐environment at and around the lesion sites, most transplantation studies have adopted the use of a cocktail of growth factors embedded in extracellular matrix or performed pre‐treatment for modulating the niche to enhance the viability and differentiation potency of the grafted cells.^[^ ^2^, ^29^, ^36^ ^]^ Notably, the intrinsic nature of human neural progenitors exhibits a prolonged time course of maturation when grafted to sites of SCI, resulting in late functional recovery of recipient animals.^[^ ^2^, ^4^ ^]^ Moreover, the beneficial effects of growth factors on the grafted cells or the niche might be transient and limited by the prolonged differentiation process. In addition, the pro‐astrocytic micro‐environment in SCI restricts the neurogenic differentiation capacity and axonal outgrowth of the grafted hNSCs.^[^ ^2^, ^3^ ^]^ These physiological features and pathological factors compromise the therapeutic potential of human grafts for severe SCI, resulting in delayed or limited functional recovery. In our study, we demonstrated that reducing the level of *SOX9* expression by half in hNSCs resulted in the activation of an intrinsic program conferring better survival, engraftment, and enhanced neurogenic potency to form new relay circuits without reliance on trophic factors support and niche modulator, which greatly improves locomotor and somatosensory functions in a severe traumatic injury model.

Functions of *SOX9* include maintaining multipotent neural stem properties, promoting astrocyte differentiation, and inhibiting neurogenesis.^[^ ^5^, ^37^ ^]^ Consistently, the pro‐astrocytic niche in SCI induces robust generation of GFAP^+^ astrocytes expressing high levels of *SOX9* that leads to the formation of glial scar.^[^ ^6^ ^]^ More importantly, over 40% of the grafted wild‐type hNSCs, which exhibited high levels of *SOX9* expression at the injury site, differentiated into astrocytes eventually. This is in line with a previous report that showed the differentiation bias of transplanted human neural progenitor cells toward astrocytes following contusion SCI.^[^ ^3c^ ^]^ Global ablation of *SOX9* in an adult mouse model of SCI showed reduced expression of genes associated with astrocytic scars and an increased level of reactive sprouting from spared axons caudal to the lesion, which promoted locomotor recovery.^[^ ^7^, ^8^ ^]^ In contrast, ablation of astrocytic scars under the control of GFAP‐Cre prevented axon regrowth and extension, even when stimulated by trophic factors together with conditioning lesions,^[^ ^24^, ^38^ ^]^ indicating the scar‐forming astrocytes aid and support spared axon regeneration. This discrepancy could be due to the studies not considering the broader impact of *SOX9* deletion not only on astrocytic scars but also on endogenous neural stem cells, which could give rise to specific neuronal cell types that restore neural connectivity to recover locomotion in SCI mice. These findings suggest that endogenous or grafted cells with persistent upregulation of *SOX9* expression in the hostile micro‐environment of SCI could result in compromised de novo neuronal regeneration,^[^ ^3c^ ^]^ implying targeting SOX9 in human neural progenitors could overcome their therapeutic limitations for the treatment of SCI. Indeed, we found that hNSCs with ≈50% reduction of *SOX9* expression exhibited high neurogenic potency with expandable capacity that coincided with the gradual diminishment of endogenous *SOX9* expression in proliferative hNSCs undergoing neuronal differentiation. Further reduction of *SOX9* expression reduced the survival and renewal capacity of hNSCs. In contrast, *SOX9*‐null embryonic NSCs remained viable but had reduced self‐renewal capacity and multipotentiality.^[^ ^5^, ^39^ ^]^ In addition, our RNA‐seq data showed that SHH expression and its downstream events were upregulated in ≈50% *SOX9 KD* hNSCs, whereas *SOX9* functions downstream of SHH to induce and maintain mouse NSCs.^[^ ^5b^ ^]^ Together, our data demonstrate that human *SOX9* has a distinct role and dosage requirement for the renewal, survival, and differentiation potency of hNSCs compared to mouse *SOX9*. The difference in *SOX9* dosage requirements between humans and mice is also evident in testis development. In humans, heterozygous mutations in *SOX9* cause skeletal defects, with ≈70% of male patients undergoing sex reversal.^[^ ^40^ ^]^ In mice, heterozygous deletion of *SOX9* resulted in normal testis development.^[^ ^41^ ^]^ Therefore, our findings together with others suggest context‐, dose‐ and species‐dependent roles of *SOX9*.

It has been shown that appropriate reduction of glycolytic metabolism is essential for cell survival and neuronal differentiation of human neural progenitors, whereas reinforced glycolysis leads to the formation of astrocytes and neuronal death.^[^ ^10^, ^42^ ^]^ Concurrently, we detected low glucose uptake and markedly reduced expression of glycolytic genes in cultured *SOX9 KD* cells, implying that *SOX9* is required for the induction and/or maintenance of high glycolytic metabolism in hNSCs. It is therefore conceivable that a potential mechanism underlying *SOX9 KD*‐enhanced neurogenesis could be mediated through a moderate reduction of glycolysis in hNSCs. In support of this notion, single‐cell transcriptomic profiling analysis showed that *SOX9* is one of the top downregulated genes in adult brain neurogenesis, which was involved in shifting energy metabolism from glycolysis to oxidative phosphorylation.^[^ ^43^ ^]^ Moreover, a few studies have reported functional associations of *SOX9* with glycolytic metabolism.^[^ ^44^ ^]^ A previous report showed that *SOX9* directly regulated UGT8 expression to promote glycolysis for the maintenance of cancer malignancy.^[^ ^44a^ ^]^ Whether *SOX9* directly or indirectly regulates UGT8 and/or other glycolytic genes in hNSCs remains to be determined. It is worth noting that *SOX9 KD* grafts were more impervious to the detrimental environment in SCI, had strong differentiation bias toward the pro‐neuronal lineage as early as 2 weeks post‐transplant, and subsequently generated substantial amounts of different neuronal subtypes compared to scramble grafts in the absence of trophic support. Importantly, *SOX9 KD* hNSCs retained low levels of glycolytic genes and high levels of neurogenic genes 2 months post‐graft following SCI, suggesting that the injury environment did not affect their metabolic state and neurogenic potency. This is further evident by the ability of *SOX9 KD* hNSCs to differentiate into neurons instead of astrocytes when challenged by the pro‐astrocytic niche at the injury site. Notably, the increased formation of oligodendrocytes from *SOX9 KD* hNSCs could be due to compensatory upregulation of SOX10 expression required for oligodendrocyte differentiation. Our data demonstrated the ability of *SOX9 KD*‐derived oligodendrocytes to myelinate own axons in the rodent host, demonstrating the superiority of *SOX9 KD* hNSCs in restoring myelinated axons at the site of injury. Previous transplantation studies using NSCs from human and rat embryonic spinal cords revealed the importance of the axial identity of grafted cells to match the anatomical site of injury in the recipient animals, which determined their viability, differentiation potency, and integration capacity for a better functional outcome. In addition, matched grafts also had ability to rewire the lesioned spinal cord.^[^ ^2^, ^35^, ^45^ ^]^ Accordingly, *SOX9 KD* grafts with spinal identity not only exhibited high neurogenic potency, but also showed good survival, uniform distribution in the lesion cavity, long‐distance axonal growth into the caudal recipient spinal cord, and the establishment of synaptic connections with host neurons, indicating its excellent integration properties. The possible reasons for the striking differences in the engraftment between scramble and *SOX9 KD* grafts could be due to the upregulated expression of SHH ligand, which likely acts in an autocrine fashion to trigger its downstream signaling effectors to promote the survival, proliferation, and motor neuron differentiation of *SOX9 KD* cells.^[^ ^46^ ^]^ Our results also suggest an inhibitory role of half *SOX9* gene dosage on SHH expression, but the underlying mechanisms remain elusive. In addition, SHH signaling could exert neuroprotective effects on the micro‐environment of CNS injuries by limiting inflammation and promoting nerve regeneration, likely via the inhibition of the production of injury‐inducing cytokines by reactive astrocytes.^[^ ^47^ ^]^ Besides SHH, *SOX9 KD* grafts also exhibited intrinsic activation of FGF8, which is not only a key factor responsible for the acquisition of spinal cord identity, but also involved in the reduction of gliosis, and the enhancement of neuronal survival and axonogenesis in SCI.^[^ ^2^, ^38^, ^48^ ^]^ The beneficial properties of *SOX9 KD* grafts were further supported by a dramatic reduction of CSPGs in the periphery,^[^ ^36^ ^]^ which enabled axonal growth beyond the lesion site, promoted regeneration of severed axons, and allowed descending and ascending fibers penetration through the graft to re‐establish the neural relay circuitry, thereby achieving better functional outcomes in restoring both locomotor and somatosensory systems.

Our work reveals a new paradigm in activating the intrinsic program by using a genetically targeted strategy to enhance the therapeutic potential of transplanted hNSCs for treating SCI. This approach alters the grafts’ response in the injury niche and confers enhanced differentiation capacity, survival, and integration, as well as reduced glial scar formation to provide a more effective stem cell therapy for severe traumatic SCI. To enable the clinical translation of our findings, it is essential to develop approaches to reduce the level of *SOX9* expression in hNSCs that will be safe for long‐term engraftment. Currently, the use of lentiviruses for *SOX9 KD* could lead to insertion mutagenesis, which poses risk of oncogenicity, toxicity, and immunogenicity. Non‐viral vectors are being developed to prevent adverse side effects; however, their transfection efficiency is significantly lower than viral carriers. Emerging therapeutics using small molecules to target transcription factor activity have received much research interest and have high clinical potential given their importance across numerous diseases.^[^ ^49^ ^]^ Previous reports have identified small‐molecule inhibitors targeting SOX18 protein‐protein interaction.^[^ ^50^ ^]^ Future efforts should focus on developing approaches to reduce the level of *SOX9* activity or expression in hNSCs by altering levels of ubiquitylation and subsequent proteasome degradation or by targeting its enhancer elements using the CRISPR‐Cas9 system, respectively.^[^ ^51^ ^]^ These innovations hold great therapeutic promise to yield hNSCs with unique cell and non‐cell autonomous properties for successfully treating SCI.

## 4 Conclusion

This study demonstrates that hNSCs with reduced *SOX9* expression by half could overcome the post‐injury milieu and intrinsic limitations with enhanced therapeutic potential for transplantation treatments for SCI.

## 5 Experimental Section

### Pluripotent Stem Cell Culture

Human pluripotent stem cells (IMR90, H9, and HES2) were provided by WiCell Research Institute (Madison, WI), and passaged 33–49. Cells were cultured in mTeSR (Stem Cell Technologies) or E8 medium (Thermo Fisher Scientific, Waltham, MA) on Matrigel (Corning)‐coated plates. Cells were passaged with ReLsR (Stem Cell Technologies), washed, and replated at a dilution of 1:10. Addition of 10 µM Y‐27632 (ROCK inhibitor; Tocris Biosciences) was applied to enhance cell survival at each passage.

### Preparation of N2B27, Neural Induction, and Neural Maintenance Medium

For N2B27 medium, 5 mL N2 supplement (100x, serum‐free), 10 mL B27 supplement (serum‐free, 100x), 10 mL MEM non‐Essential amino acids (100x), 5 mL penicillin–streptomycin, 5 mL Glutamax, were aseptically mixed and made up to 500 mL in DMEM/F12 medium. All reagents were purchased from Thermo Fisher Scientific. To prepare 10 mL of neural induction medium, 10 mL N2B27 medium supplemented with 250 nM LDN‐193189 (Tocris Biosciences) were aseptically mixed in DMSO, 10 µM SB‐431542 in DMSO, 4 µM CHIR‐99021 (Tocris Biosciences) in DMSO, 200 ng mL^−1^ SHH (R&D) and 250 nM RA (Tocris Biosciences). For neural maintenance medium, N2B27 medium was supplemented with 20 ng mL^−1^ EGF and 20 ng mL^−1^ FGF (Peprotech, Rocky Hill, NJ). For neuronal differentiation medium, N2B27 medium was supplemented with 20 ng mL^−1^ BDNF, 20 ng mL^−1^ GDNF, 20 ng mL^−1^ IGF, 100 ng mL^−1^ SHH, and 250 nM RA (first 2 days). For neural medium for assaying scramble and *SOX9 KD* spontaneous differentiation capacity, N2B27 medium was supplemented with 300 ng mL^−1^ cAMP (Sigma‐Aldrich) and 0.2 mM vitamin C (Sigma‐Aldrich) without any other trophic factors.

### Neural Induction, Maintenance, and Neurosphere Culture

Human pluripotent stem cells were suspended on a low attachment plate for 2 days in a neural induction medium, and then plated on Matrigel‐coated plates to induce PAX6‐ and SOX2‐expressing neuroepithelia (NE) formation in neural induction medium as previously described.^[^ ^16^ ^]^ The medium was replaced with fresh neural induction media every 2 days. The neuroepithelial cells started to form neural tube‐like rosettes on days 7–9, which were gently blown off using a 200 µL pipette and replaced in matrigel‐coated plate on day 10. At 10 days after neural induction, the neural induction media was replaced with neural maintenance media, and cells were split 1:3 on an Accutase plate with Matrigel‐coated wells for subsequent passaging. Addition of 10 µM Y‐27632 (ROCK inhibitor; Tocris Biosciences) was applied to enhance cell survival at each passage. For the neurosphere assay, neural stem cells (NSCs) at passage 1 were dissociated into a single‐cell suspension at 10^5^ mL^−1^ and transferred onto 6‐well low attachment plates (Corning). Neurospheres were cultured in neural maintenance medium to 3 mL in each well. For secondary neurosphere formation, primary neurospheres were further dissociated into a single‐cell suspension after 7 days, and 10^5^ mL^−1^ of the cell suspension was transferred into 6‐well low attachment plates.

### Constructs and Cell Lines

shRNA was designed against human *SOX9 KD*1 (5’‐GACTTCGGCAACGTGGACATT‐3′) and *SOX9 KD2* (5’‐GAGACACCATCTACCAAGTTT‐3′) based on the previously published study^[^ ^52^ ^]^ and using the principles from The RNAi Consortium (https://www.broadinstitute.org/rnai/public/) and then cloned them into lentiviral vector pLKO.1‐puro or eGFP or Tet‐PLKO‐neo. The pLKO.1‐TRC control was a gift from David Root (Addgene plasmid #10879). The human *SOX9* cDNA was cloned into lentiviral vector pLVX‐EF1*α*‐puro (Clontech).^[^ ^52^ ^]^ For lentivirus production, 5 × 10^6^ 293 T cells were plated in a 100‐mm dish and transfected with a lentiviral expression vector, packaging plasmid psPAX.2, and envelope plasmid pMD2.G using PolyJet (SignaGen). The cell culture medium containing the lentiviral particles was harvested at 48 and 72  h post‐transfection, filtered through a 0.45‐µm filter, and analyzed by flow cytometry for GFP expression to calculate viral titer. The lentiviral particles were titrated by Lenti‐X qRT‐PCR Titration Kit (Takara). Next, 3 × 10^5^ human pluripotent stem cells were infected with lentivirus particles expressing cDNA and/or shRNA and cultured in the presence of 8 µg mL^−1^ Polybrene (Sigma) for 24  h. After 48  h transduction, infected cells were screened in presence of 1 µg mL^−1^ puromycin (Life Technologies) or 1.0 mg mL^−1^ neomycin. Dox dissolved in DMSO was added to a culture medium <1% without causing cell death based on previous studies.^[^ ^53^ ^]^

### Clonal Analysis

Single GFP‐positive hiPSCs or its derived hNSCs were expanded and enriched by a BD FACSAria IITM fushion cell sorter. Briefly, the cell suspension was washed in DMEM and filtered through a 50‐µm nylon cell strainer. The single‐cell suspension was collected by centrifugation and suspended in neural maintenance media and then sorted by FACSAria II at the imaging and flow cytometry core in Centre for PanorOmic Sciences of the University of Hong Kong. GFP‐positive sorted cells were further expanded for 4‐6 weeks (up to four passages for hNSCs) before grafting or qPCR analysis.

### Electrophysiology

Recordings were made in a submersion‐type recording chamber and perfused with oxygenated artificial cerebrospinal fluid containing 119 mM NaCl, 2.5 mM KCl, 2 mM MgCl_2_, 2.5 mM CaCl_2_, 1.3 mM NaH2PO_4_, 26.0 mM NaHCO_3_, and 20 mM glucose (≈295 mOsml) at 23 °C at a rate of 2–3 mL min^−1^. Whole‐cell patch‐clamp recordings were obtained using Multiclamp 700B patch amplifiers (Molecular Devices), and data were analyzed with pClamp 10 software (Molecular Devices). Data were low‐pass filtered at 2 kHz and digitized at 10 kHz. Whole‐cell voltage and current‐clamp recordings were made at room temperature with pulled patch pipettes (5–6 m) filled with an internal solution containing 150 mM K‐gluconate, 1.5 mM MgCl_2_, 5.0 mM HEPES, 1 mM EGTA, 10 mM phosphocreatine, 2.0 mM ATP, and 0.3 mM GTP. Voltage‐gated sodium and potassium channels were blocked with 250 µM and 1 mM tetrodotoxin (Tocris Biosciences, Minneapolis, MN).

### RNA Sequencing

Total RNA was collected with the RNeasy mini kit from six samples from the indicated groups and stored at −80 °C. RNA integrity was examined with the Agilent Bioanalyzer 2000 (Agilent, BGI). TrueSeq stranded mRNA‐seq libraries were prepared from 1 µg of total RNA (Illumina mRNA‐seq kit, RS‐122‐2103) and sequenced on an Illumina HiSeq 2500 at the IGM Genomics Center (BGI). Reads were mapped to human genome GRCh38 using the STAR spliced read aligner with default parameters. Between 88.5% and 92.3% (average: 90.0%) of the reads were mapped uniquely to the human genome. Total counts of read fragments aligned to candidate gene regions were derived using the HTSeq program (http://htseq.readthedocs.io). Expression levels of the subtype cluster genes were quantified in reads per kilobase of transcript per million mapped reads (RPKM) and visualized on the UCSC Genome Browser. Next, RPKM values were obtained as described above and used for unsupervised hierarchical clustering.

### Animals

Sprague–Dawley male rats (230–260 g) from the Centre for Comparative Medicine Research, Li Ka Shing Faculty of Medicine, The University of Hong Kong were used. All animal protocols were approved by Committee on the use of live animals in Teaching & Research (CULATR 4905–18) of The University of Hong Kong. Health guidelines for laboratory animal care and safety were strictly followed. Animals had free access to food and water throughout the study.

### SCI Contusion Surgery (Severe)

Sprague–Dawley male rats were anesthetized with an intraperitoneal injection of ketamine (80 mg kg^−1^) and xylazine (10 mg kg^−1^) mixture. A laminectomy was conducted at the caudal portion of T6 and at all T7 spinal levels. A T7‐8 severe contusion SCI (weight of 25 g, height of 50 mm) was produced with a modified version of MASCIS impactor. After performing the spinal contusion, muscle, and skin layers were sutured with 4.0 polyglactin. The bladder of each injured animal was squeezed manually twice a day after SCI for 2 to 3 weeks.

### Cell Engraftment

Fourteen days after SCI surgery, animals underwent a second procedure for cell implantation. Rats were anesthetized with an intraperitoneal injection of ketamine (80 mg kg^−1^) and xylazine (10 mg kg^−1^) mixture. The original incision was reopened and the injury sites were re‐exposed. To investigate the survival and spontaneous differentiation capacity of scramble and *SOX9 KD* hNSCs, Scramble and *SOX9 KD* hNSCs were re‐suspended in DMEM/F12 medium supplemented with 10 µM Rock Inhibitor at a density of 10^5^ cells µL^−1^ without growth factors. Cells were kept on ice throughout the procedure. Two injections were performed at the injury site, each delivering 1.5 µL of the cell suspension at −0.5 and +0.5 mm distance from dorsal middle line (0 mm). The injection was performed using a 30 G syringe (Hamilton) connected with a micropump (RWD), with the animals tightly fixed in a stereotaxic apparatus (RWD). The rate of the injection was 250 nL min^−1^. The syringe was left in place for an additional 10 min before and after the injections. At the end of the procedure, the muscle and skin layers were sutured with 4.0 polyglactin and animals received subcutaneous injections of buprenorphine (0.03 mg/kg) and meloxicam (2 mg kg^−1^) for 3 days, and oral administration of enrofloxacin (2.5%) for 7 days. Cyclosporine (5 mg kg^−1^) was subcutaneously injected every day for immune‐suppression. Animals underwent functional testing for up to 16 weeks and were sacrificed for the anatomical analysis by transcardial perfusion with 4% formaldehyde.

### Anterograde Labeling of the CST

Three weeks before sacrifice, descending CST fibers of grafted rats were labeled with BDA (10% BDA in 0.9% saline, molecular probes) by injecting into five spots of the right motor cortex. The skulls of anesthetized rats (ketamine (80 mg kg^−1^) and xylazine (10 mg kg^−1^) were tightly fixed to a stereotaxic apparatus (RWD). A vertical midline incision was made from between the eyes to the posterior skull. The injection area on the right hemisphere defined in a rectangle measuring 2 mm (from 1.0 mm anterior to −1.0 mm posterior to the bregma) by 1.5 mm (lateral to the bregma). A drill was used to create the injection sites on the skull. Injections were performed using a 33G syringe (Hamilton) attached to a micropump (RWD). Each injection delivered 100 nL of the BDA solution into the motor cortex at a rate of 100 nL min^−1^. The injector tip was left in place for an additional 5 min before and after the injections. For visualization, BDA staining was performed by incubating the sections in 3% H_2_O_2_ for 30 min to reduce endogenous peroxidase, followed by 2 h incubation with streptavidin–horseradish peroxidase (Vectastain R.T.U. Elite ABC Reagent, Cat. No: PK‐7100) at room temperature. PerkinElmer Biotinyltyramide (1:100 in amplification diluent) was then added to the sections for another 1 h. Detection was accomplished by incubation with Extra‐Avidin@ TRITC (1:200, Sigma) in 0.1% PBS‐T for 2 h.

### Retrograde Tracing

AAV2‐Retro‐Syn‐mCherry was purchased from the BrainVTA. Rats were anesthetized by ketamine/ xylazine and the lumbar spinal column was exposed by incision of the skin and blunt dissection of muscles. The T9 vertebrae were used as a landmark to identify the L1 and L2 vertebrae. Rats were mounted in a custom spine stabilizer. AAV particles(2.0 × 10^12 Vg/ml^) were injected into the spinal cord through a 33G syringe (Hamilton) driven by a micropump (RWD) and guided by a micromanipulator (pumping rate: 150 nL min^−1^). Injections were made between L1 and L2 vertebrae, bilaterally, 0.5 mm lateral to the midline, 0.75 mm, and 1.5 mm depth, 0.5 ul/injection site. Animals were transcardially perfused with 0.9% saline and 4% paraformaldehyde solutions in 0.1 M  PB after 2 weeks of injection. Brains, brainstem, and spinal cords were dissected and fixed overnight in 4% paraformaldehyde at 4 °C followed by 2 days 30% sucrose. After embedding, transverse and sagittal sections were gathered for subsequent analysis. The emitted mCherry fluorescence from the sections was strong enough to image without the need to amplify by immunofluorescence.

### Histology and Immunohistochemistry

Animals were euthanized by intraperitoneal injection of pentobarbitone overdose (150–200 mg kg^−1^) before perfusing with 0.9% saline followed by 4% PFA. Spinal cords were removed, post‐fixed, and sectioned at 20‐80 µm intervals. Sections were dried overnight at room temperature. Sections were then incubated with primary antibodies overnight (Table S1, Supporting Information) followed by incubation with Alexa Fluor 488, 594, or 647–conjugated donkey secondary antibodies (1:500; Invitrogen) for 1 h at room temperature. For nuclear staining, DAPI was added to the final wash. Images were captured under confocal microscopy (LSM 800 or 900, Zeiss) using 10×, 20×, or 40×(oil) objectives using Z‐stack or tiles.

### Quantification of Corticospinal axons, 5‐HT labeled Axons or Neural Cell Type in Grafts

For quantification of neural differentiation or growth in human cell grafts, eight to nine randomly selected fields of grafts from six to eight animals per group were visualized using a Carl Zeiss LSM 800 or 900 confocal microscope at a magnification of 100× or 200×. The proportion of neural‐, neuronal‐, or glial marker‐expressing cells relative to the total number of GFP‐ or HuNu‐ or DAPI‐expressing cells was calculated and averaged among groups. The number of corticospinal axons regenerating into grafts in the lesion sites was quantified as previously described.^[^ ^54^ ^]^ In brief, one in six sections(80 µm thickness) was stained for 5‐HT or BDA‐labeled CST axons. Using ZEN offline, dorsal‐to‐ventral virtual lines were placed at regular distances under 100× magnification, and graft/host interface and the number of axons intercepting were examined under 200× magnification. 5‐HT or BDA‐labeled axons that intersected the line were marked and counted.

### Quantification of Glial Scars

Sections fluorescently immunolabeled for GFAP or CSPG and GFP were used to quantify the intensity of the field occupied by GFAP or CSPG immunoreactive areas surrounding the graft‐lesion cavity and lesion cavity alone, as previously described.^[^ ^3b^ ^]^ A 100‐µm wide zone surrounding the lesion site was quantified in this manner.

### Behavioral Studies for Locomotor Activity

A total of 129 rats underwent SCI and were randomized to receive scramble or *SOX9 KD* grafts in different batches. 16 out of 126 rats died during surgery or after injury or were eliminated during the experiment due to unsuccessful SCI based on the BBB scores. Eight rats that received scramble NSC grafts were removed from the study due to the survival of a few cells in the absence of growth factors. The BBB open‐field 21‐point locomotion rating scale was performed in the weekly assessments of the remaining rats, conducted by two independent observers to confirm successful SCI.^[^ ^55^ ^]^ To investigate survival and early differentiation, rats were sacrificed at 2 weeks (6 scrambles and 6 *SOX9 KD*), 3 weeks (4 scrambles and 4 *SOX9 KD*), and 1 month (9 scrambles and 10 *SOX9 KD*) post graft. Next, BDA injections were administered in rats sacrificed at 2 months (10 scrambles and 10 *SOX9 KD*), and retrograde virus injections were administered in rats sacrificed at 3 months post‐injury (15 scrambles and 15 *SOX9 KD*).

Grid walk assessment was performed using a modified grid (4×6 cm grids), and hind limb foot drops were recorded as a measure of hind limb sensorimotor function. Two investigators blinded to group identity assessed outcomes. Foot drops were recorded if the rat was unable to grasp a grid rung with a hind paw during stepping and paw placement, which resulted in a foot drop below the grid. Percentage of total paw replacements = Total steps–foot drops/Total steps (including left and right hind limb). Testing was performed on uninjured rats prior to surgery as a baseline measurement, and then every 2 to 3 weeks post‐injury. Three trials per rat were evaluated and the scores were averaged for the analysis. For the foot fault scoring, a qualitative analysis was performed on skilled walking as previously described.^[^ ^30^ ^]^ Briefly, the score was defined as follows: correct placement = 6 points; partial placement = 5 points; placement correction = 4 points; replacement = 3 points; slight slip = 2 points; deep slip = 1 point; and total miss = 0 points. With the limb that started the walk, consecutive steps were then estimated. Three trials per rat were evaluated and the scores were averaged for the analysis. For the footprint analysis, animals were placed on 1 m long narrow corridor lined with force sensors that recorded the pressure exerted by the feet due to locomotor behavior, which was converted into a digital image of plantar surface, which reflects the hind limb supporting ability and coordination. Rats were then allowed to run three times along the corridor, and stereotyped gait and motor coordination parameters, including hind limb stride length and width were measured from three complete step cycles from the middle of the runway.^[^ ^26^ ^]^

### Behavioral Tests for Sensation

The mechanical nociceptive threshold was assessed using the von Frey test. Briefly, rats were placed individually in transparent plastic boxes on a stainless‐steel mesh‐bottomed platform for 30 min to acclimate to the environment. Hind paw withdrawal threshold (PWT), which responded to blunt von Frey filaments connected to a calibrated electronic von Frey filament esthesiometer (IITC Life Science Inc, Woodland Hill, CA), was recorded automatically by the esthesiometer. The filaments were applied perpendicularly to the plantar surface of the ipsilateral hind paw (surgery side). Quick withdrawal (but not due to locomotion) or licking of the paw was considered a positive response. Three repeated measurements were made per animal under each test session with a 3‐min interval.

Cold avoidance was assayed by two temperature preference tests (Ugo Basile, Italy). Rats were allowed to acclimate on the plate at ambient temperature (20 °C ± 2 °C) for 60 min. At the end of the habituation period and 2 h before “warm‐up,” the temperature‐controlled plates were turned on at two different temperatures, with one side of the plate set to 30 °C ± 1 °C and the other side at ambient temperature (left schematic). The locomotor activity of the rat was recorded by a portable digital camera and analyzed by Smart 3.0 video tracking software. Given the learning and memory ability of rats, the temperature settings between the reference plate and test plate were interchanged for the next trial. Each rat was tested twice for each set of parameters and rats were re‐habituated between trials.

The Radiant Heat Test was conducted on rats individually placed in a plastic box with a transparent glass platform. Thermal hyperalgesia of bilateral hind paw was measured by Hargreaves Apparatus (Ugo Basile, Varese, Italy) using a radiant heat source under the glass platform in contact with the plantar surface of hind paw. To avoid tissue damage, the cut‐off time was set to 20 s. Hind paw withdrawal latency(PWL) was recorded for each hind paw and averaged over three repeated measurements. In each session, one right paw and one left paw were stimulated at 10 min intervals. Each rat was tested three times and the mean was regarded as the PWL.^[^ ^56^ ^]^

### Statistical Analysis

For comparison between two groups two‐tailed Student's t‐test was used at a designated significance level of *p < 0.05*. The normality assumption was verified using the Shapiro‐Wilk test. Measurements taken at different time points were compared using one‐way repeated‐measures ANOVA or two‐way repeated‐measures ANOVA, followed by Tukey post hoc or Bonferroni test. Gene expression data were analyzed using two‐tailed one‐sample Student's *t‐*tests when compared to baseline control group. Statistical analyses were performed using Prism 8 (GraphPad Software) with a designated significance level of 95%. All data were presented as mean ± SEM. The statistical details of each experiment could be found in the figure legends. No statistical methods were used to calculate sample size estimates.

## Conflict of Interest

The authors declare no conflict of interest.

## Author Contributions

J.AI.L., K.W.T., K.L‐K.W., D.K‐Y.S., Y‐S.C., and M.C. designed the experiments; J.AI.L., K.W.T., Y.L.C., X.L.F., C.W.L.C., A.L.H.L., K.L‐K.W., M‐N.H., M‐H.W., K.K‐K.C., and M.P.L.C. performed the experiments; J.AI.L., K.W.T., Y.L.C., K.L‐K.W., M‐N.H., and M.C. analyzed and interpreted the results; J.AI.L., C.W.C., D.K‐Y.S., Y‐S.C., and M.C. supervised the project; J.AI.L., and M.C. wrote the manuscript.

## Supporting information

Supporting Information

Supplemental Table 1

## Data Availability

The data that support the findings of this study are available in the supplementary material of this article.
