# Supplementary material for: Transplanting Human Neural Stem Cells with ≈50% Reduction of SOX9 Gene Dosage Promotes Tissue Repair and Functional Recovery from Severe Spinal Cord Injury
Source: Adv Sci (Weinh). 2023 Jun 9;10(20):2205804. doi: 10.1002/advs.202205804 (PMC10369238; doi:10.1002/advs.202205804)
Supplement: Supplementary file 1 — Supporting Information [file ADVS-10-2205804-s002.pdf]

ADVANCED SCIENCE
Open Access

Supporting Information

for *Adv. Sci.*, DOI 10.1002/advs.202205804

Transplanting Human Neural Stem Cells with ≈50% Reduction of *SOX9* Gene Dosage Promotes Tissue Repair and Functional Recovery from Severe Spinal Cord Injury

Jessica Aijia Liu, Kin Wai Tam, Yong Long Chen, Xianglan Feng, Christy Wing Lam Chan, Amos Lok Hang Lo, Kenneth Lap-Kei Wu, Man-Ning Hui, Ming-Hoi Wu, Ken Kwok-Keung Chan, May Pui Lai Cheung, Chi Wai Cheung, Daisy Kwok-Yan Shum, Ying-Shing Chan and Martin Cheung**

Supporting information

Transplanting human neural stem cells with approximately 50% reduction of *SOX9* gene dosage promotes tissue repair and functional recovery from severe spinal cord injury

Jessica Aijia Liu, Kin Wai Tam, Yong Long Chen, Xianglan Feng, Christy Wing Lam Chan, Amos Lok Hang Lo, Kenneth Lap-Kei Wu, Man-Ning Hui, Ming-Hoi Wu, Ken Kwok-Keung Chan, May Pui Lai Cheung, Chi Wai Cheung, Daisy Kwok-Yan Shum, Ying-Shing Chan and Martin Cheung**

Supporting Experimental Section

Immunocytochemistry

Cultures were fixed for 30 min in 4% paraformaldehyde (PFA) in 0.1 M phosphate buffer at 4°C. After washing three times with PBS, fixed samples were permeabilized with 0.1% Triton X-100 with 1% BSA in PBS for 1 h at room temperature. Primary antibodies in blocking solution were added and incubated overnight at 4°C. The primary antibodies used for the immunolabeling are listed in Table S1. After rinsing, samples were incubated with donkey Alexa Fluor–conjugated secondary antibodies (1:500; Invitrogen) for 1 h at room temperature. Nuclear counterstaining was carried out with 4′,6-diamidino-2-phenylindole (DAPI). Images were captured by a confocal microscope (LSM 800 or 900, Zeiss) using 20× or 40× (oil) objective.

Quantification of cells in vitro.

Marker-positive and total cells (DAPI) were quantified by ImageJ software combined with manual counting for MAP2, GFAP, and TUJ1. One day after splitting, samples were fixed and stained. Next, four to six images from left to right, and then up and down were captured at 200× or 400× magnification.

Quantitative reverse-transcription polymerase chain reaction

Total RNA was isolated from the culture with the RNeasy mini kit (Qiagen, Hilden, Germany) according to the manufacturer's protocol. Total RNA was quantified with NanoDrop (Thermo Fisher). For cDNA synthesis, the reverse-transcription reaction was carried out with PrimeScript RT master mix (Perfect Real Time, Clontech) and quantitative PCR was performed using primers specific for the genes of interest (list in Table S2) with SYBR Premix Ex Taq II

(Tli Rnase H Plus; Clontech) in 20-μL reactions volumes. We used the ΔΔCt method to calculate the relative gene expression with Actin as the internal control.

Western blotting

Cells were washed twice in cold phosphate-buffered saline (PBS) and lysed in RIPA buffer (150 mM NaCl, 1 mM EDTA, 1% NP40, 0.5% Sodium deoxycholate, 0.1% SDS, 50 mM Tris-HCl, pH 7.5) supplemented with 1% protease and phosphatase inhibitor cocktail (Thermo Fisher). Proteins were separated by SDS-PAGE using a Bio-Rad system under reducing conditions. Membranes were probed with antibodies against SOX9 (H-90, Santa Cruz or Millipore), and Actin overnight at 4 °C and then incubated with appropriate horseradish peroxidase-conjugated goat anti-rabbit (at 1:2000, Dako) at room temperature for 1 h. After incubation with the ECL substrate for 1–3 min, blots were exposed to X-ray film (FujiFilm Super RX) at different times to obtain the optimal intensity of the protein bands and images were analyzed by ImageJ.

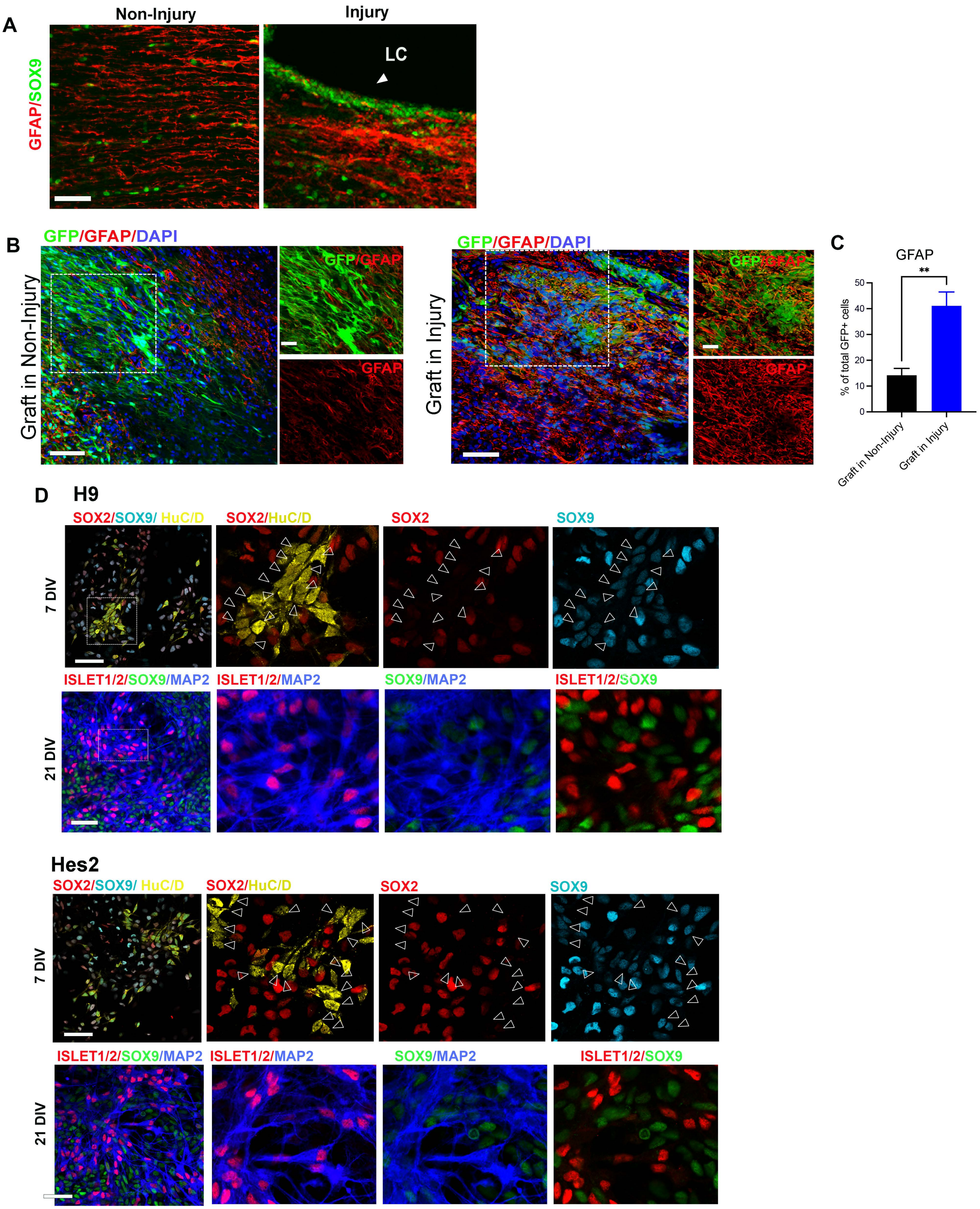
A
Non-Injury
Injury
GFAP/SOX9
LC
B
GFP/GFAP/DAPI
Graft in Non-Injury
GFP/GFAP
GFAP
GFP/GFAP/DAPI
Graft in Injury
GFP/GFAP
GFAP
C
GFAP
**
% of total GFP+ cells
50
40
30
20
10
0
Graft in Non-Injury
Graft in Injury
D
H9
SOX2/SOX9/ HuC/D
SOX2/HuC/D
SOX2
SOX9
7 DIV
ISLET1/2/SOX9/MAP2
ISLET1/2/MAP2
SOX9/MAP2
ISLET1/2/SOX9
21 DIV
Hes2
SOX2/SOX9/ HuC/D
SOX2/HuC/D
SOX2
SOX9
7 DIV
ISLET1/2/SOX9/MAP2
ISLET1/2/MAP2
SOX9/MAP2
ISLET1/2/SOX9
21 DIV

Figure S1. SCI induces SOX9 expression in both endogenous NSCs and grafted hNSCs which tend to differentiate into astrocytes instead of neurons. (**A**) Representative immunofluorescence images of SOX9 and GFAP in spinal cord sagittal sections of sham and injury groups at 14 days post-injury. The white arrow indicates cystic lesion cavity (LC). Scale bar, 200μm. (**B**) Representative images show that GFP+ grafts in the spinal cords differentiate into astrocytic (GFAP+) fates at 2 months after grafting in non-injury and injury group. White box shows a magnified view with indicated markers. Scale bar for the magnified view, 200μm; scale bar for lower power view, 50μm. (**C**) Quantification of GFAP+ in GFP+ grafts. means ± SEM, n = 5; Student t-test $^{**}p< 0.01$. (**D**) Representative immunofluorescence images for SOX2, SOX9, and HuC/D in hNSCs derived from H9 and Hes2 at 7 days differentiation (DIV) and for MAP2, SOX9, and ISLET1/2 after 21 DIV. The empty arrowheads indicate the differentiating neuronal cells without SOX2 expression but exhibit moderate reduction of SOX9 expression. Scale bar, 50 μm.

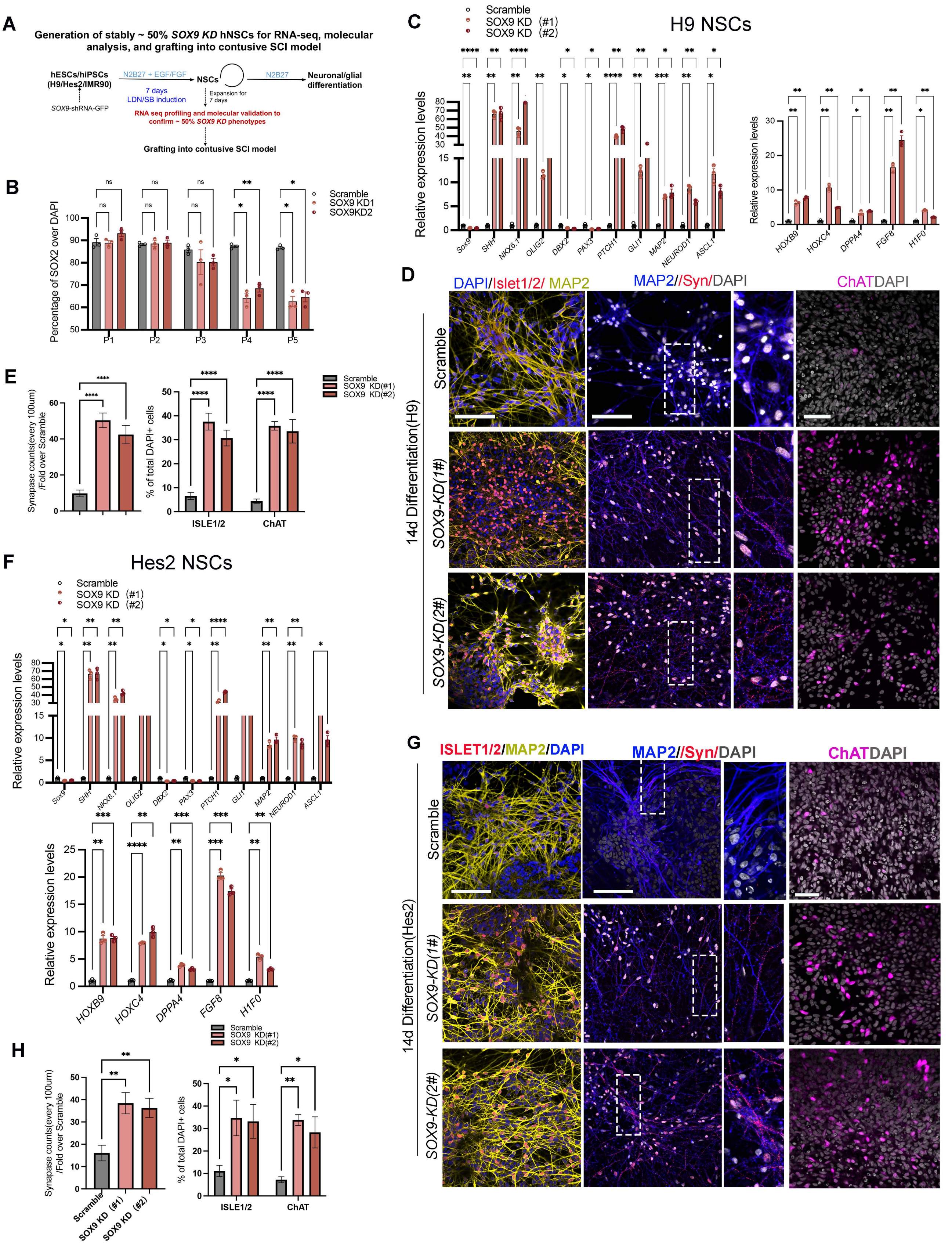
A
Generation of stably ~ 50% SOX9 KD hNSCs for RNA-seq, molecular analysis, and grafting into contusive SCI model
hESCs/hiPSCs (H9/Hes2/IMR90)
SOX9-shRNA-GFP
N2B27 + EGF/FGF
7 days
LDN/SB induction
NSCs
Expansion for 7 days
N2B27
Neuronal/glial differentiation
RNA seq profiling and molecular validation to confirm ~ 50% SOX9 KD phenotypes
Grafting into contusive SCI model
B
Scramble
SOX9 KD1
SOX9KD2
Percentage of SOX2 over DAPI
P1
P2
P3
P4
P5
C
H9 NSCs
Scramble
SOX9 KD (#1)
SOX9 KD (#2)
Relative expression levels
Sox9
SHH
NKX6.1
OLIG2
DBX2
PAX3
PTCH1
GLI1
MAP2
NEUROD1
ASCL1
HOXB9
HOXC4
DPPA4
FGF8
H1F0
D
DAPI/Islet1/2/ MAP2
MAP2//Syn/DAPI
ChATDAPI
Scramble
SOX9-KD(1#)
SOX9-KD(2#)
14d Differentiation(H9)
E
Synapase counts(every 100um) /Fold over Scramble
% of total DAPI+ cells
ISLE1/2
ChAT
Scramble
SOX9 KD(#1)
SOX9 KD(#2)
F
Hes2 NSCs
Scramble
SOX9 KD (#1)
SOX9 KD (#2)
Relative expression levels
Sox9
SHH
NKX6.1
OLIG2
DBX2
PAX3
PTCH1
GLI1
MAP2
NEUROD1
ASCL1
HOXB9
HOXC4
DPPA4
FGF8
H1F0
G
ISLET1/2/MAP2/DAPI
MAP2//Syn/DAPI
ChATDAPI
Scramble
SOX9-KD(1#)
SOX9-KD(2#)
14d Differentiation(Hes2)
H
Scramble
SOX9 KD(#1)
SOX9 KD(#2)
Synapase counts(every 100um) /Fold over Scramble
% of total DAPI+ cells
ISLE1/2
ChAT
Scramble
SOX9 KD (#1)
SOX9 KD (#2)

Figure S2. Validation of maker genes expression from RNA-sequencing in the scramble and *SOX9 KD* hNSCs derived from H9 and Hes2

(**A**) Experimental procedure for the generation of stably ~50% SOX9 KD hNSCs for RNA-seq profiling, molecular analysis, and grafting into contusive SCI rodent model. (**B**) Quantification of $SOX2^+$ cells in neurospheres treated with scramble control and *SOX9 KD* from passages 1 to 5. (**C**) qPCR analysis of markers associated with neural tube patterning, neuronal differentiation, and spinal markers in H9-derived NSCs. (**D**) Representative immunofluorescence images for MAP2, ISLET1/2, ChAT, and synaptophysin (Syn) in the scramble and *SOX9 KD* hNSCs derived from H9 at 14 days post-differentiation. White box shows a magnified view with the indicated markers (Scale bar, 50 μm). (**E**) Quantification of markers (**D**). (**F**) qPCR analysis of markers associated with neural tube patterning, neuronal differentiation, and spinal markers in Hes2-derived NSCs. (**G**) Representative immunofluorescence images for MAP2, ISLET1/2, ChAT, and Syn in the scramble and *SOX9 KD* hNSCs derived from Hes2 at 14 days post-differentiation. White box shows a magnified view with the indicated markers (Scale bar, 50 μm). (H) Quantification of markers in (**G**). All data are expressed as mean ± SEM. *p<0.01, **p<0.05, ***p<0.005 versus scramble. Three independent experiments. Student t-test.

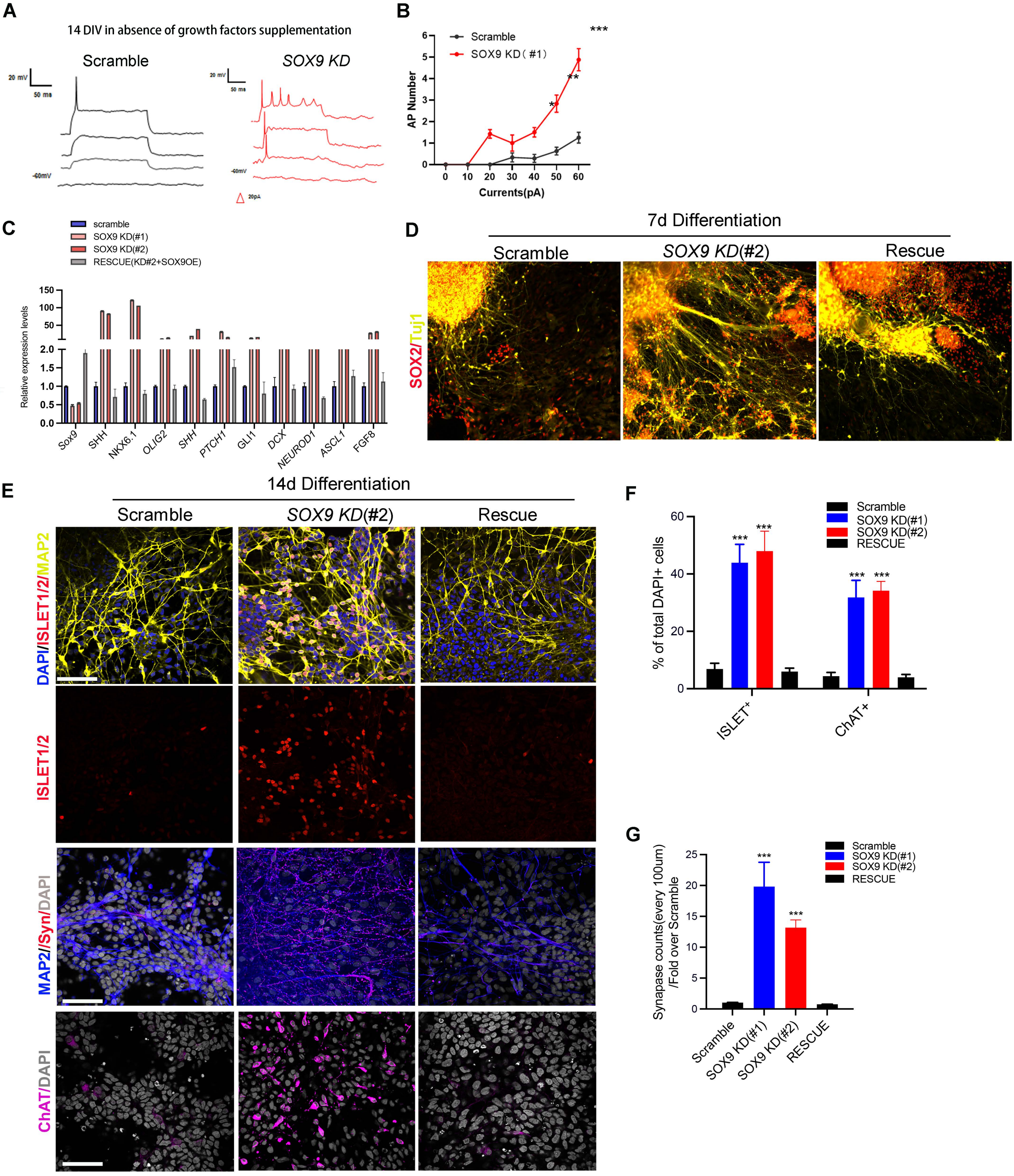
A
14 DIV in absence of growth factors supplementation
Scramble
SOX9 KD
B
Scramble
SOX9 KD(#1)
AP Number
Currents(pA)
C
scramble
SOX9 KD(#1)
SOX9 KD(#2)
RESCUE(KD#2+SOX9OE)
Relative expression levels
Sox9
SHH
NKX6.1
OLIG2
SHH
PTCH1
GLI1
DCX
NEUROD1
ASCL1
FGF8
D
7d Differentiation
Scramble
SOX9 KD(#2)
Rescue
SOX2/Tuj1
E
14d Differentiation
Scramble
SOX9 KD(#2)
Rescue
DAPI/ISLET1/2/MAP2
ISLET1/2
MAP2/Syn/DAPI
ChAT/DAPI
F
Scramble
SOX9 KD(#1)
SOX9 KD(#2)
RESCUE
% of total DAPI+ cells
ISLET+
ChAT+
G
Scramble
SOX9 KD(#1)
SOX9 KD(#2)
RESCUE
Synapase counts(every 100um)
/Fold over Scramble

Figure S3. *SOX9 KD* hNSCs exhibit enhanced neurogenic potency with fate bias towards motor neurons.

(A) Representative traces showing responses to depolarizing current injection (0 pA; 20 pA; 40 pA; 60 pA) from a resting potential of −60 mV in scramble- and SOX9 KD-derived neurons 14 days DIV. (B) The amplitude of evoked action potentials. (C) qPCR analysis of markers associated with SHH signaling, neural tube patterning, and neuronal differentiation. One-way ANOVA followed by Tukey's post-hoc test. (D) Representative immunofluorescence images for SOX2, TuJ1, and DAPI of hNSCs treated with scramble control, *SOX9 KD* (#2), and rescue constructs at 7 days differentiation. (E) Representative images for MAP2, ISLET1/2, Syn, ChAT and DAPI of hNSCs treated with scramble control, SOX9 KD (#2), and rescue constructs at 14 days differentiation. (F) Percentage of ISLET1/2+ and ChAT+ in different treatment groups. (G) Quantification of synapses in different treatment groups. Student t-test. All data are expressed as mean ± SEM.*$p<0.05$,**$p<0.01$, ***$p<0.005$. Three independent experiments.

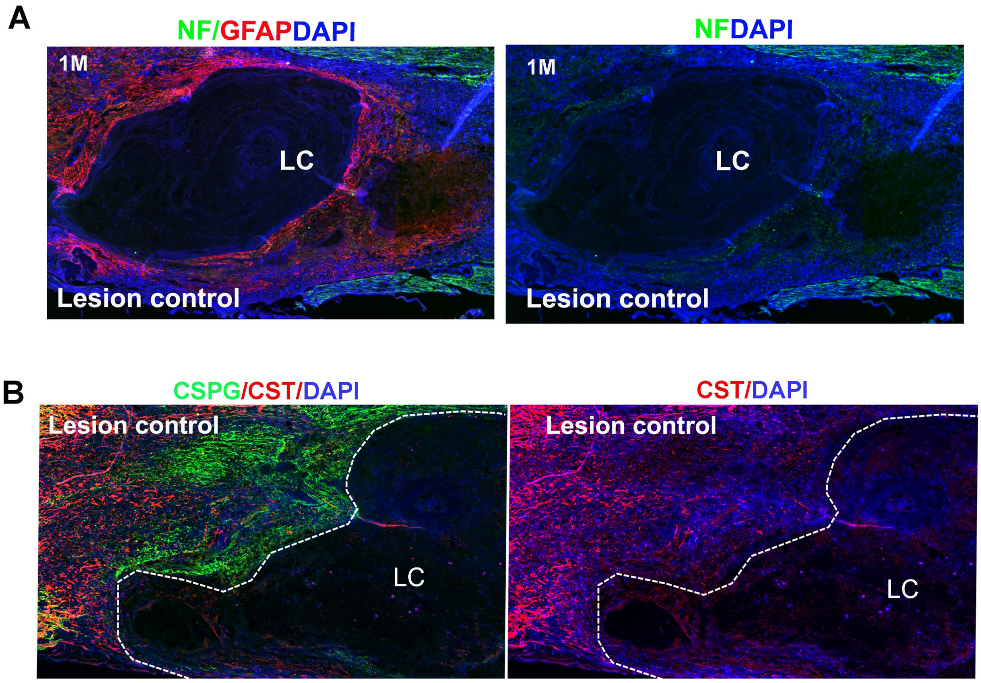
A
NF/GFAPDAPI
NFDAPI
1M
LC
Lesion control
B
CSPG/CST/DAPI
CST/DAPI
Lesion control
LC

Figure S4. SCI-induced lesion cavity surrounded by glial scar to prevent the regrowth of axon and CST. (**A**) Representative immunofluorescence images for GFAP and neurofilament (NF) on longitudinal sections of rat spinal cord lesion control (n=6). LC, lesion cavity. (**B**) Representative images of CST fibers and CSPG expression in the periphery of the LC. Dotted lines outline the LC.

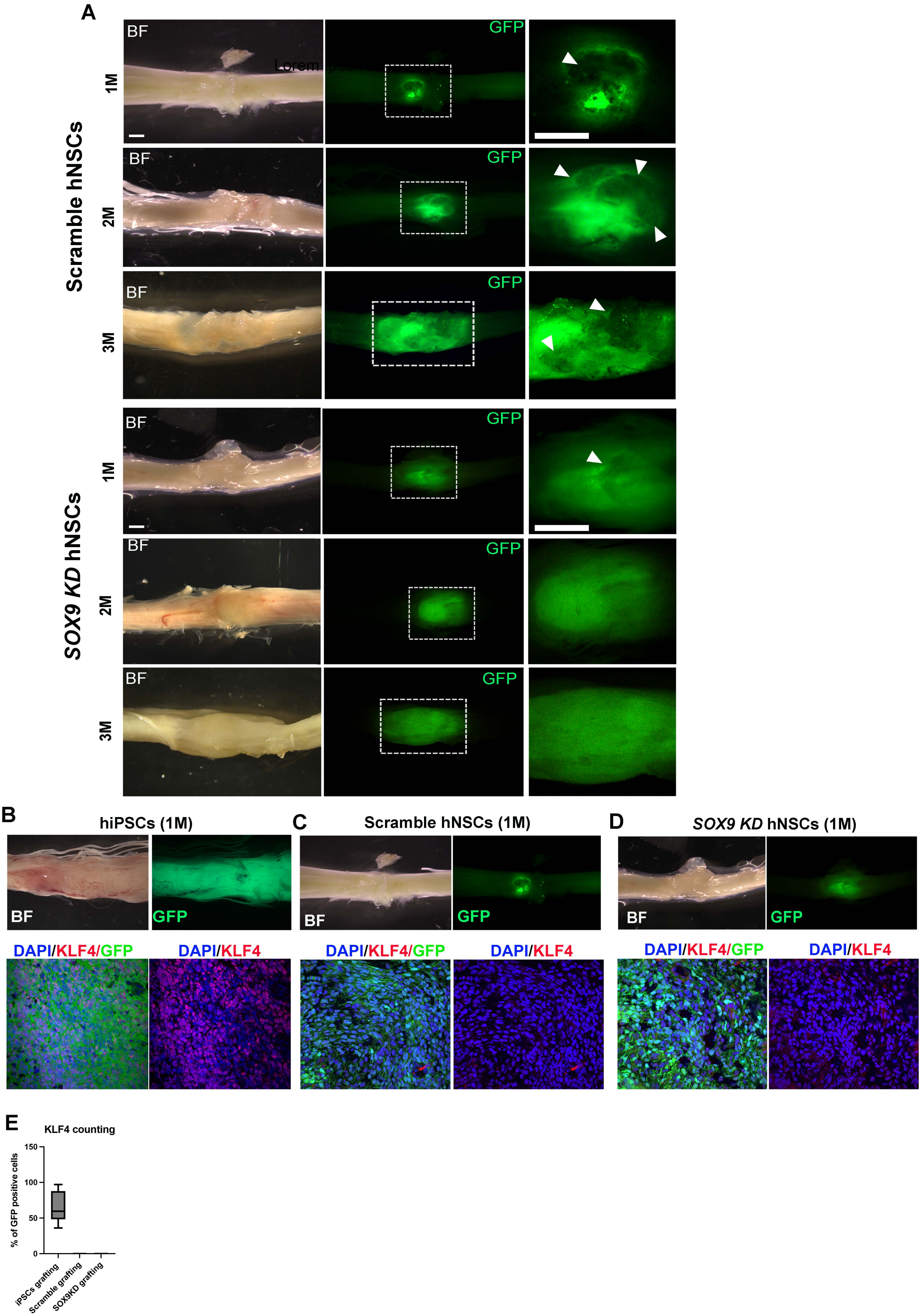
A
BF
GFP
1M
2M
3M
Scramble hNSCs
SOX9 KD hNSCs
B
hiPSCs (1M)
C
Scramble hNSCs (1M)
D
SOX9 KD hNSCs (1M)
DAPI/KLF4/GFP
DAPI/KLF4
E
KLF4 counting
% of GFP positive cells
150
100
50
0
iPSCs grafting
Scramble grafting
SOX9KD grafting

Figure S5. **In the absence of growth factor, *SOX9 KD* grafts exhibit efficient distribution into the lesion cavity and integration with the host tissue of SCI model without tumor formation. (A)** Longitudinal view of spinal cord showing the distribution of GFP-labeled NSCs treated with the scramble and *SOX9 KD* implanted into the site of injury at 1, 2, and 3 months post-graft (n=6 per treatment for each time point). White boxes show a high-magnified view. White arrows indicate the lesion cavity of the injured spinal cord. Scale bar: 5 μM. Bright field (BF) and fluorescence view of contusive SCI grafted with GFP+ iPSCs (**B**), scramble hNSCs (**C**) and *SOX9 KD* hNSCs (**D**) at 1 month (M) post-injury. Representative immunofluorescence images for GFP, KLF4, and DAPI on longitudinal spinal cord sections. (**E**) Quantification of KLF4+ cells in the indicated treatment group.

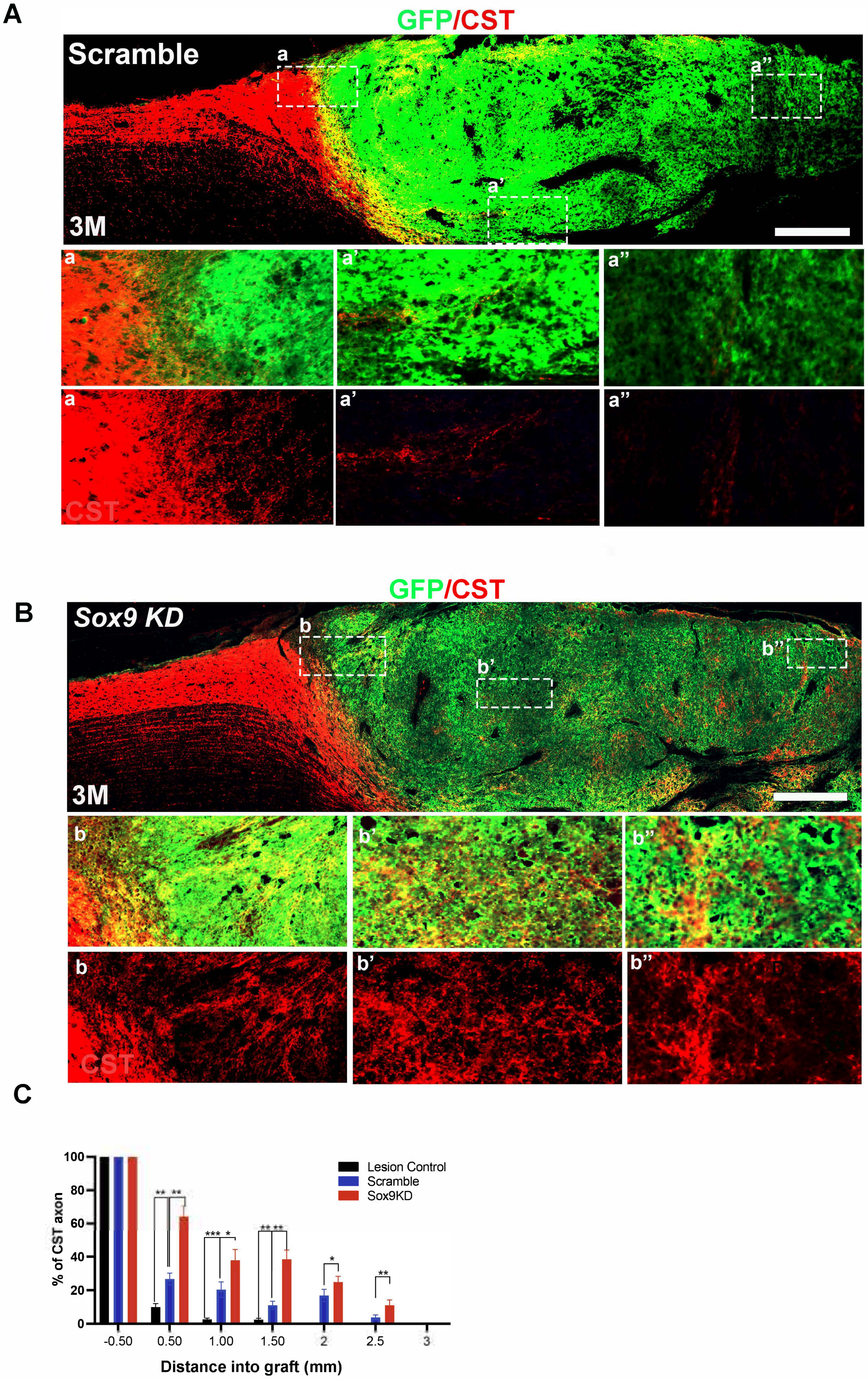
A
GFP/CST
Scramble
3M
a
a'
a''
CST
B
GFP/CST
Sox9 KD
3M
b
b'
b''
CST
C
% of CST axon
Distance into graft (mm)
Lesion Control
Scramble
Sox9KD
100
80
60
40
20
0
-0.50
0.50
1.00
1.50
2
2.5
3

Figure S6. ***SOX9 KD* grafts promote innervation of the cortical spinal fiber (CST) tract.** Representative images of CST+ fibers extending into the scramble (A) and *SOX9 KD* GFP+ grafts. (n=6 per treatment) (B). a-a'' and b-b'' show a high magnified view of CST+ fibers innervating into the graft at different regions from rostral to caudal (Scale bar = 100 μM). Quantification of the distance of CST axons innervating into scramble and SOX9 KD grafts, normalized to the total number of CST axons 0.5 mm rostral to the lesion site (n=4). One-way ANOVA. * $p < 0.01$, ** $p < 0.05$. All data are presented as mean ± SEM.

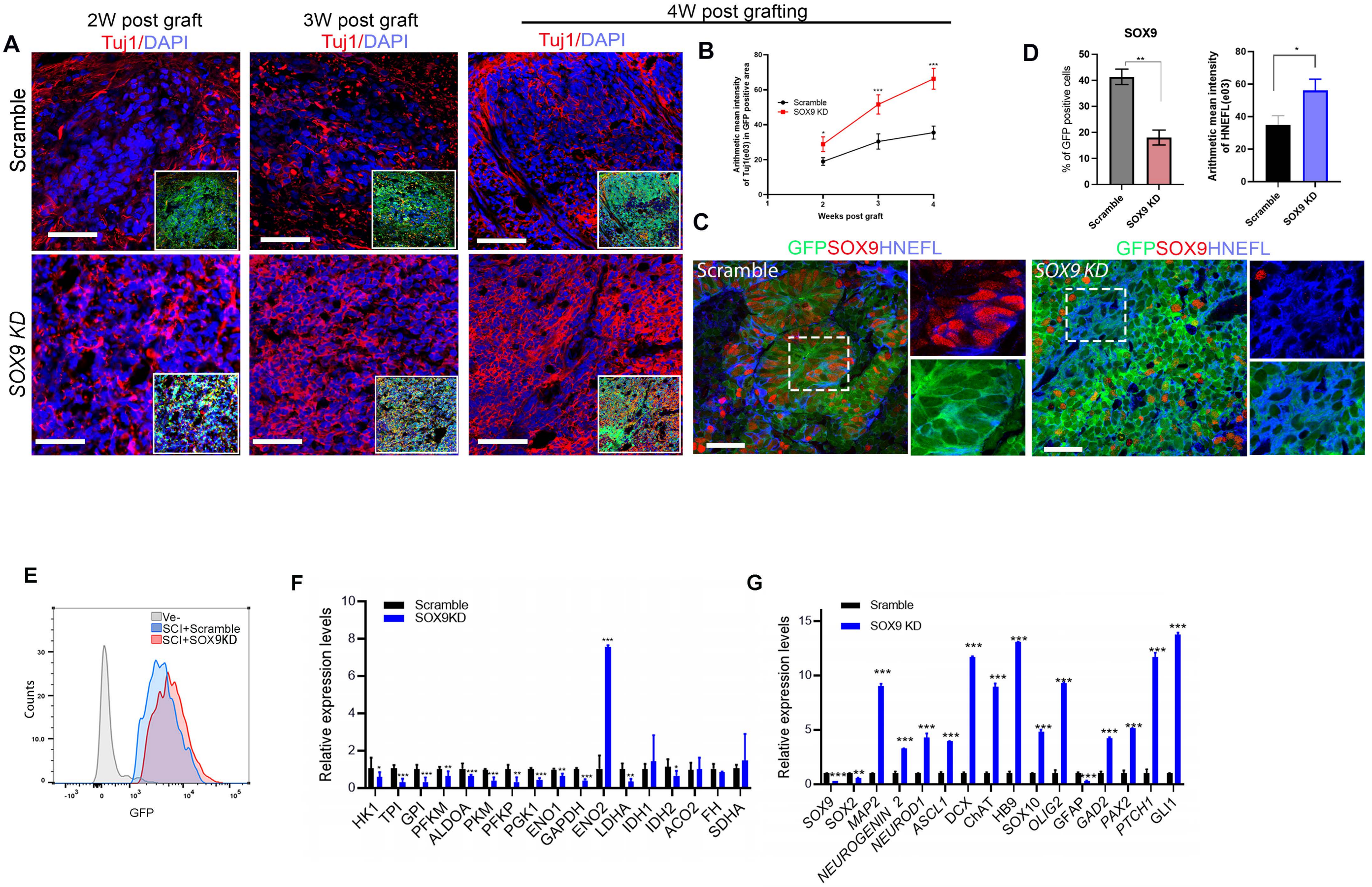

A
2W post graft
3W post graft
4W post grafting
Tuj1/DAPI
Scramble
SOX9 KD
B
Arithmetic mean intensity of Tuj1(e03) in GFP positive area
Scramble
SOX9 KD
Weeks post graft
C
GFPSOX9HNEFL
Scramble
SOX9 KD
D
SOX9
% of GFP positive cells
Arithmetic mean intensity of HNEFL(e03)
E
Ve-
SCI+Scramble
SCI+SOX9KD
Counts
GFP
F
Relative expression levels
Scramble
SOX9KD
HK1
TPI
GPI
PFKM
ALDOA
PKM
PFKP
PGK1
ENO1
GAPDH
ENO2
LDHA
IDH1
IDH2
ACO2
FH
SDHA
G
Relative expression levels
Sramble
SOX9 KD
SOX9
SOX2
MAP2
NEUROGENIN 2
NEUROD1
ASCL1
DCX
ChAT
HB9
SOX10
OLIG2
GFAP
GAD2
PAX2
PTCH1
GLI1

Figure S7. ***SOX9 KD* hNSCs retain high neurogenic potency and low glycolysis at the site of SCI. (A)** Representative immunofluorescence images of the time course (2, 3, and 4 weeks (W) post-graft) analysis of neuronal differentiating marker (Tuj1) in the injured cord grafted with the scramble and SOX9 KD hNSCs. Insets showing the distribution of GFP-labeled hNSCs implanted into the site of the injury (Scale bar,100 µm). (**B**) Fluorescence intensity analysis of Tuj1 in the hNSC graft at different time points (n = 4 rats per each time point). One-way ANOVA. *p<0.05, ***p<0.005. (**C**) Representative immunohistochemical images for GFP (green), SOX9 (red), and HNEFL (Blue, human-specific neurofilament) in the injured spinal cord grafted with the scramble and *SOX9 KD* hNSCs 4 weeks post-graft. White boxes show a zoomed-in view with indicated markers (Scale bar, 50 µm). (**D**) Quantification of SOX9+ and fluorescence intensity analysis of HNEFL in the indicated treatment group (n = 4 per group). (**E**) Representative flow images showing the GFP-positive cell counts sorted from negative control, spinal cord injury (SCI) grafted with scramble hNSCs, and *SOX9 KD* hNSCs at 2M post-graft. (**F**) qPCR analysis of key glycolysis and TCA cycle metabolic genes from sorted grafted GFP+ hNSCs treated with the scramble and *SOX9 KD*. (**G**) qPCR analysis of neural progenitor markers (*SOX2*, *SOX9*), neuronal differentiation markers (*MAP2, NEUROGENIN2, NEUROD1, ASCL1, DCX1*), subtypes of neuronal markers (*ChAT, HB9, SOX10, OLIG2, GFAP, GAD2, PAX2*) and HH signaling (PTCH1, GLI1). *p<0.05,**p<0.01,***p<0.005. All data were presented as mean ± SEM.

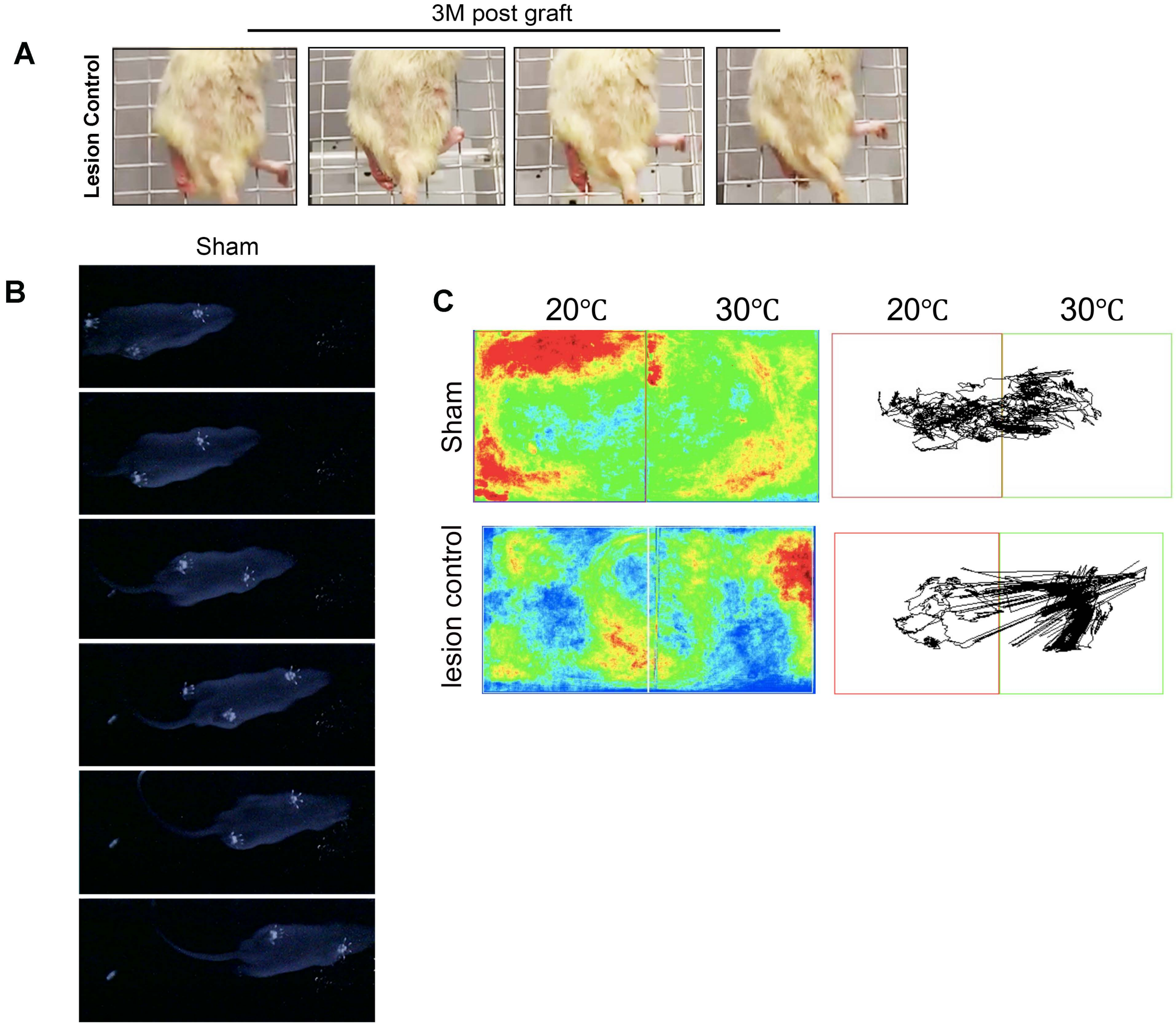
A
3M post graft
Lesion Control
Sham
B
C
20℃
30℃
20℃
30℃
Sham
lesion control

Figure S8. Lesion control. (A) Representative images showing foot placement during ladder rung walking in SCI rats (lesion control) without grafting hNSCs after 14 weeks of injury (3 months post-grafting). (B) Representative video images showing footprints of sham control. (C) Representative track imaging of the two-plate preference test at 30^0C versus 20^0C in sham and SCI lesion control at 14 weeks post-injury

Antibody	Manufacturer	Catalog # / clone	Species
Sox9	Merck Millipore	AB5535	Rabbit
Sox9	H-90, Santa Cruz	sc-25574	Rabbit
Sox10	R & D systems	AF2864	Goat
Sox2	R & D systems	AF2018	Goat
Sox2	R & D systems	MAB2018	Mouse
Sox2	Abcam	ab97959	Rabbit
Sox1	R & D systems	AF3369	Goat
Islet1/2	Developmental Studies Hybridoma Bank	39.4D5	mouse
Nkx6.1	Developmental Studies Hybridoma Bank	F55A12-c	mouse
HB9	Developmental Studies Hybridoma Bank	81.5C10	mouse
Olig2	Millipore	Ab9610	Rabbit
HuC/D	Invitrogen Antibodies	A-21271	Mouse
NGN2	R & D systems	MAB3314	Rabbit
Pax6	Developmental Studies Hybridoma Bank	aa 1-223	mouse
Pax6	Life Technologies	1557865A	Rabbit
HuNu (Human)	Millipore	MAB1281 / 235-1	Mouse
Ki67	Abcam	ab15580	Rabbit
caspase-3	Abcam	Ab2302	Rabbit
Tuj1	R & D systems	MAB1195	Mouse
MAP2	Abcam	ab32454	Rabbit
MAP2	Abcam	ab11267	Mouse
5-HT	ImmunoStar	20079	Goat

CaMKII	Abcam	ab52476	Rabbit
ChAT	Millipore	AB144P	Goat
GABA	Sigma	A2052	Rabbit
NF70 (Human)	Millipore	MAB5294	Mouse
NG2 (Human)	Millipore	MAB2029	Mouse
GFAP	Dako	Z0334	Rabbit
GFAP	Abcam	Ab53554	Goat
Synaptophysin(human)	Novus Biologicals	NBP1-19222	Mouse
Synaptophysin	Developmental Studies Hybridoma Bank	SV2A	Mouse
GFP	GeneTex	GTX13970	Chicken
MBP	Millipore	AB980	Rabbit

Table S1: List of antibodies used for immunofluorescence

Gene symbol	**Forward**	**Reverse**
HK2	CTTCTTCACGGAGCTCAACC	AAGCCCTTTCTCCATCTCCT
PGK	GCCAAGTCGGTAGTCCTTATG	CCCAGCAGAGATTTGAGTTCTA
PKM	CTGTGGCTGGACTACAAGAA	CTGCTTCACCTGGAGAGAAATA
FPKM	GCATCCCATTTGTGGTCATTC	GTCACAGGTTGTGCAGATAGT
PFKP	GATGACTCAGGATGTGCAGAAG	CGCTTGTAGGTGTTCAGGTT
ENO1	GTGCAGGAATCCAGGTAGTG	TGGTTGACTTTGAGCAGGAG
ENO2	CTGATCCTTCCCGATACATCAC	CTGGTCAAATGGGTCCTCAA
PGAM1	ACCTGGAGAACCGCTTCAG	TCGCTCTCTTCTGCACTGAG
IDH2	GAGCACCAAGAACACCATACT	CGGTGCTCATACCAGATCTTATT
SDHA	AGAGGGAGGCATTCTCATTAAC	ACCGAGACACCACATCTCTA
IDH1	TGAAGAAGGTGGTGGTGTTG	GATACAAAGGCCAACCCTTAGA
ALDOA	GTGTCATCCTCTTCCATGAGAC	AACACCGCCCTTGGATTT
LDHA	GAAGACTCTGCACCCAGATTTA	CCAATAGCCCAGGATGTGTAG
HK1	CATGACAACCCACTGAGAAGAG	CAGCCAACGAAGGTCAGATT
ACO2	GCCCAACGAGTACATCCATTA	ATCCAGGTGTCCATACACAATC
OGDH	CTGCCCTCAAGACCATCATT	ACATTTGCAAGCACGTTCAG
Sucla2	GTGAGCGAAAATATCCCAGG	TTGCTTCAGGAGACTCAGCA
FH	GGAGGTGTGACAGAACGCAT	CCTCATCTGCTGCCTTCATT
CS	CTCCCTTTCTTACCTCCCCA	GGGCTGCAAGAACAAGACA
SDHB	AAGGCTGGAGACAAACCTCA	CACAGATGCCTTCTCTGCAT
SOX9	ACACACAGCTCACTCGACCTTG	GGAATTCTGGTTGGTCCTCTCTT
NKX2.2	GAGTCACCGGACAATGACAA	GTCTGCGCCTTGGAGAAA
NKX6.1	GAAGAGGACGACGACTACAATAAG	CTGCTGGACTTGTGCTTCT
Olig2	CAGTGGCTTCAAGTCATCCT	GCTCCGGCTCTGTCATTT
Olig3	GGAGAGAGCAGCAAGTACAAA	CTAGGTTCAGGTCGTGCATC
DBX2	TCCAACAGGTGTATCCAAGAAG	GTCCCATATTGAAGGACATGGA
PAX3	AAGAGGAAACAGCGCAGAAG	GGCCAGTTCCTCCCTAGTATAA
PTCH1	ACCGACACACACGACAATAC	ACAGTGGACTGCATGGTAATC
GLI1	GCAGTAAAGCCTTCAGCAATG	GCCAGGGAGCTTACATACATAC
MAP2	AAACTGCTCTTCCGCTCAGACACC	GTTCACTTGGGCAGGTCTCCACAA
NEUROD1	GGTGGTGCCTTGCTATTCTAA	AAAGCGTCTGAACGAAGGAG
ASCL1	TTGGTCAACCTGGGCTTT	CAGTGTCTCCACCTTACTCATC
DCX	CCATTGATGGATCCAGGAAGAT	TGACAGACCAGTTGGGATTG
LHX2	TGTTTCAGCAAGGACGGTAG	TATCACCTCAACTGCTTCACG
LIM2	GACAGCATCGCATACTGGAA	GATGAGCGAAGGCCATGAT
GAD2	GCTCTGGCGATGGGATATTT	AGCCATTCCTTTCTCCTTGAC
MSX2	GCCAAGACATATGAGCCCTAC	GGAGCTGGGATGTGGTAAAG
36B4	GTGATGTGCAGCTGATCAAGACT	GAAGACCAGCCCAAAGGAGA

Table S2: PCR primers used in the study
